# Cellular ATP redistribution achieved by deleting *Tgparp* improves lignocellulose utilization of *Trichoderma* under heat stress

**DOI:** 10.1186/s13068-024-02502-8

**Published:** 2024-04-18

**Authors:** Tuo Li, Yang Liu, Han Zhu, Linhua Cao, Yihao Zhou, Dongyang Liu, Qirong Shen

**Affiliations:** 1 Key Lab of Organic-Based Fertilizers of China and Jiangsu Provincial Key Lab for Solid Organic Waste Utilization, Nanjing, China; 2 College of Resources & Environmental Sciences, Nanjing Agricultural University, Nanjing, 210095 Jiangsu China

## Abstract

**Background:**

Thermotolerance is widely acknowledged as a pivotal factor for fungal survival across diverse habitats. Heat stress induces a cascade of disruptions in various life processes, especially in the acquisition of carbon sources, while the mechanisms by which filamentous fungi adapt to heat stress and maintain carbon sources are still not fully understood.

**Results:**

Using *Trichoderma guizhouense*, a representative beneficial microorganism for plants, we discover that heat stress severely inhibits the lignocellulases secretion, affecting carbon source utilization efficiency. Proteomic results at different temperatures suggest that proteins involved in the poly ADP-ribosylation pathway (*Tg*PARP and *Tg*ADPRase) may play pivotal roles in thermal adaptation and lignocellulose utilization. *Tg*PARP is induced by heat stress, while the deletion of *Tgparp* significantly improves the lignocellulose utilization capacity and lignocellulases secretion in *T. guizhouense*. Simultaneously, the absence of *Tgparp* prevents the excessive depletion of ATP and NAD^+^, enhances the protective role of mitochondrial membrane potential (MMP), and elevates the expression levels of the unfolded protein response (UPR)-related regulatory factor *Tgire*. Further investigations reveal that a stable MMP can establish energy homeostasis, allocating more ATP within the endoplasmic reticulum (ER) to reduce protein accumulation in the ER, thereby enhancing the lignocellulases secretion in *T. guizhouense* under heat stress.

**Conclusions:**

Overall, these findings underscored the significance of *Tgparp* as pivotal regulators in lignocellulose utilization under heat stress and provided further insights into the molecular mechanism of filamentous fungi in utilizing lignocellulose.

**Supplementary Information:**

The online version contains supplementary material available at 10.1186/s13068-024-02502-8.

## Background

The *Trichoderma* genus represents one of the most widely distributed beneficial microbes in the global agricultural system, engaging in robust interactions with plant roots and soil microbes [1]. Nowadays, *Trichoderma* has been extensively utilized as a common bio-agent, playing a crucial role in promoting plant growth and controlling soil-borne diseases [2], thus greatly contributing to advancements in agricultural production. During the colonization process in diverse habitats, including soil and root systems, *Trichoderma* typically acquires sufficient energy resources for growth by secreting lignocellulases to decompose dead fungi or plant debris [3]. Furthermore, *Trichoderma* can adapt to local environmental changes and compete with other organisms by secreting lignocellulases, thereby occupying new ecological niches [4]. In recent years, the characteristic of *Trichoderma* secreting a large number of lignocellulases has been extended to the field of chemical engineering, facilitating the processing of agricultural waste and the development of green fuels [5, 6]. Therefore, in-depth research into the mechanism of *Trichoderma* secreting cellulases, aiming to maximize lignocellulases production, holds significant importance for promoting *Trichoderma* colonization and the industrial application of enzymes.

The regulation of lignocellulases secretion primarily involves three mechanisms, including direct transcriptional regulation, upstream regulation through nutritional sensing pathways, and feedback regulation from the secretion pathway [7]. Despite the involvement of different regulatory modes, the fine-tuning cooperation of transcription factors (TFs) appears to be relatively conserved among various filamentous fungi [8]. Currently, five main classes of TFs have been identified in filamentous fungi, primarily including the positive regulators XYR1, ACE2, and the HAP2/3/5 complex, as well as the repressor protein ACE1 and the carbon catabolite repression inhibitor CRE1 [9]. The post-translational regulation of cellulase is another extensively studied mechanism. Following transcription and translation, newly synthesized lignocellulases are transported to the endoplasmic reticulum (ER) for sorting and processing before secretion. This targeting may lead to an increased protein flux in the ER, resulting in heightened demands for protein folding, disulfide bond formation, glycosylation, and sorting, consequently causing significant ER stress and unfolded protein response (UPR) [10, 11]. In *S. cerevisiae*, the UPR activated HAC1 by initiating IRE1, and the efficiently translated HAC1 enhanced the transcription of a set of genes, thereby strengthening ER folding capacity and protein transport [12]. During the degradation of lignocellulose, the scarcity of favored carbon sources (such as sugars) can also induce ER stress and UPR in filamentous fungi [7]. However, there still needs more in-depth research on how to alleviate ER stress and UPR to sustain the secretion of cellulolytic enzymes.

Temperature, one of the most ubiquitous environmental factors, regulates many physiological and morphological processes in fungi [13]. Optimal temperature is crucial for fungal growth and development, and an increase in temperature generally leads to attenuation and ultimately results in organism death [14]. Due to global warming, the sustained increase in surface temperature has various adverse effects on soil microbes, including the colonization of *Trichoderma* in the soil [15]. On the other hand, as an efficient cellulolytic fungus and beneficial plant symbiont [16], *Trichoderma* is widely used in China for solid-state fermentation of crop straw to produce biofertilizers [17]. However, the stacking and sealing of substrates, coupled with the enhanced metabolic activity of fungi, typically result in a rapid increase in system temperature. Excessive temperatures significantly limit the biomass of *Trichoderma*. Moreover, precise temperature control and human intervention significantly increase costs. Therefore, enhancing the ability of *Trichoderma* to degrade lignocellulose under heat stress becomes particularly important.

Conventional wisdom suggests that one of the most effective adaptation mechanisms for filamentous fungi under heat stress is the heat stress response (HSR). This gene expression regulatory circuit, which is highly conserved, induces the expression of a series of cytoprotective genes that encode heat shock proteins (HSPs) [18]. In addition, maintaining energy homeostasis by preserving ATP levels is imperative because many pivotal proteins are ATP-dependent under heat stress [19]. For example, protein folding, refolding, and the UPR, which limit the accumulation of unfolded proteins in the ER, are all energy-requiring processes. Hence, the question arises as to whether there is a potential mechanism in filamentous fungi that links heat stress to the lignocellulases secretion, possibly by maintaining ATP homeostasis or alleviating ER stress. In this study, employing enzymatic assays, omics analyses, and genetic manipulations, we provided further evidence to demonstrate that *Trichoderma* could alleviate ER stress and enhance lignocellulases secretion by elevating cellular ATP levels. This study is expected to provide a better understanding of the lignocellulose catabolism mechanism of filamentous fungi and provide more references for exploring lignocellulase regulatory networks.

## Results

### Evaluation of lignocellulases secretion at different temperatures in *Trichoderma guizhouense*

**Fig. 1:**
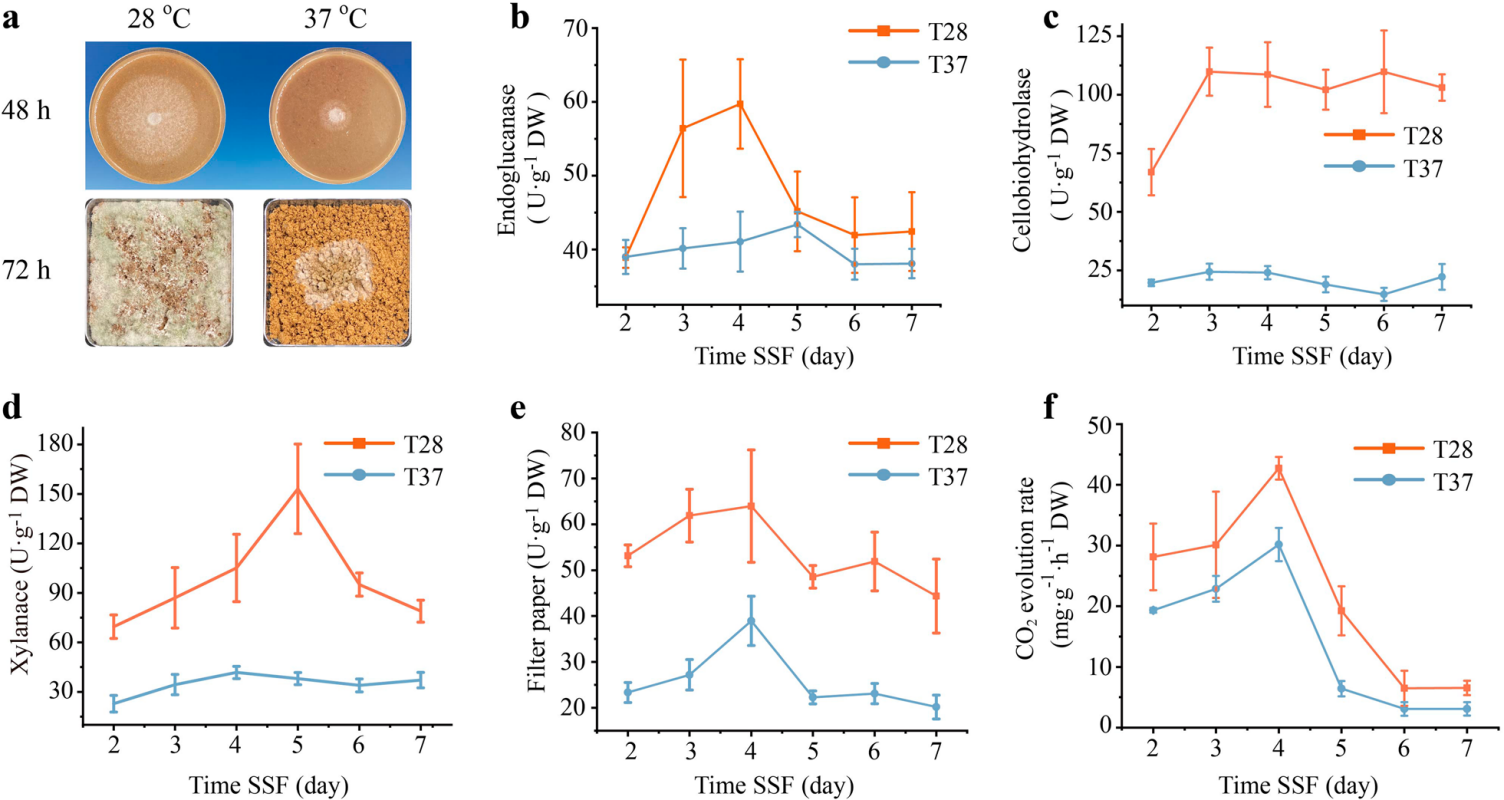
Characterization of the lignocellulose utilization efficiency of *T. guizhouense* at different temperatures.** a** Growth of *T. guizhouense* using rice straw as the sole carbon source at 28 °C and 37 °C. **b-e** Lignocellulase activities, including endoglucanase, cellobiohydrolase, xylanase, and filter paper activities, between T28 and T37 under SSF. **f** Released amount of carbon dioxide (CO_2_ evolution rate) of *T. guizhouense* in T28 and T37 under SSF. The results are presented as the mean of at least three replicates, and the bars indicate the standard error of replicates

Temperature is a critical parameter influencing the growth and metabolism of microbes. We studied the carbon utilization efficiency of *Trichoderma guizhouense* (*T. guizhouense*) at various temperatures, revealing an optimal growth temperature around 28 °C, with significant inhibition observed above 35 °C using rice straw as the sole carbon source (Additional file 1: Fig. S1). Based on this phenotype, 28 °C and 37 °C were selected as representative temperatures to investigate the relationship between lignocellulases secretion and heat stress (Fig. 1a). The systematic assessment of various lignocellulases activities is the preferred method for determining fungal lignocellulases secretion level [20]. Therefore, different lignocellulases, including endoglucanase, cellobiohydrolase, xylanase, and filter paper activity (FPA), were determined at 28 °C (T28) and 37 °C (T37) during solid-state fermentation (SSF). As expected, different lignocellulases activities exhibited consistent trends during continuous SSF, showing significant reductions in T37 compared to T28, indicating lower lignocellulose utilization efficiency in *T. guizhouense* at heat stress (Fig. 1b–e). The CO_2_ evolution rate is a critical parameter to evaluate the growth and metabolism condition of soil fungi [21]. Not surprisingly, the maximum respiration rate occurred on the fourth day, with a close to 30% decrease in T37 compared to T28 (Fig. 1f). Besides, SEM was also employed to assess the extent of lignocellulose biodegradation, and the result indicated that the degradation of rice straw was significantly lower in T37, with almost no exposure of the lower thick-walled tissue compared to T28 (Additional file 1: Fig. S1). These findings suggested that the ability to secrete lignocellulases was severely inhibited in *T. guizhouense* under heat stress, thereby limiting the efficiency of carbon sources utilization and metabolic rates.

### Quantitative analysis of the proteomes at different temperatures

**Fig. 2:**
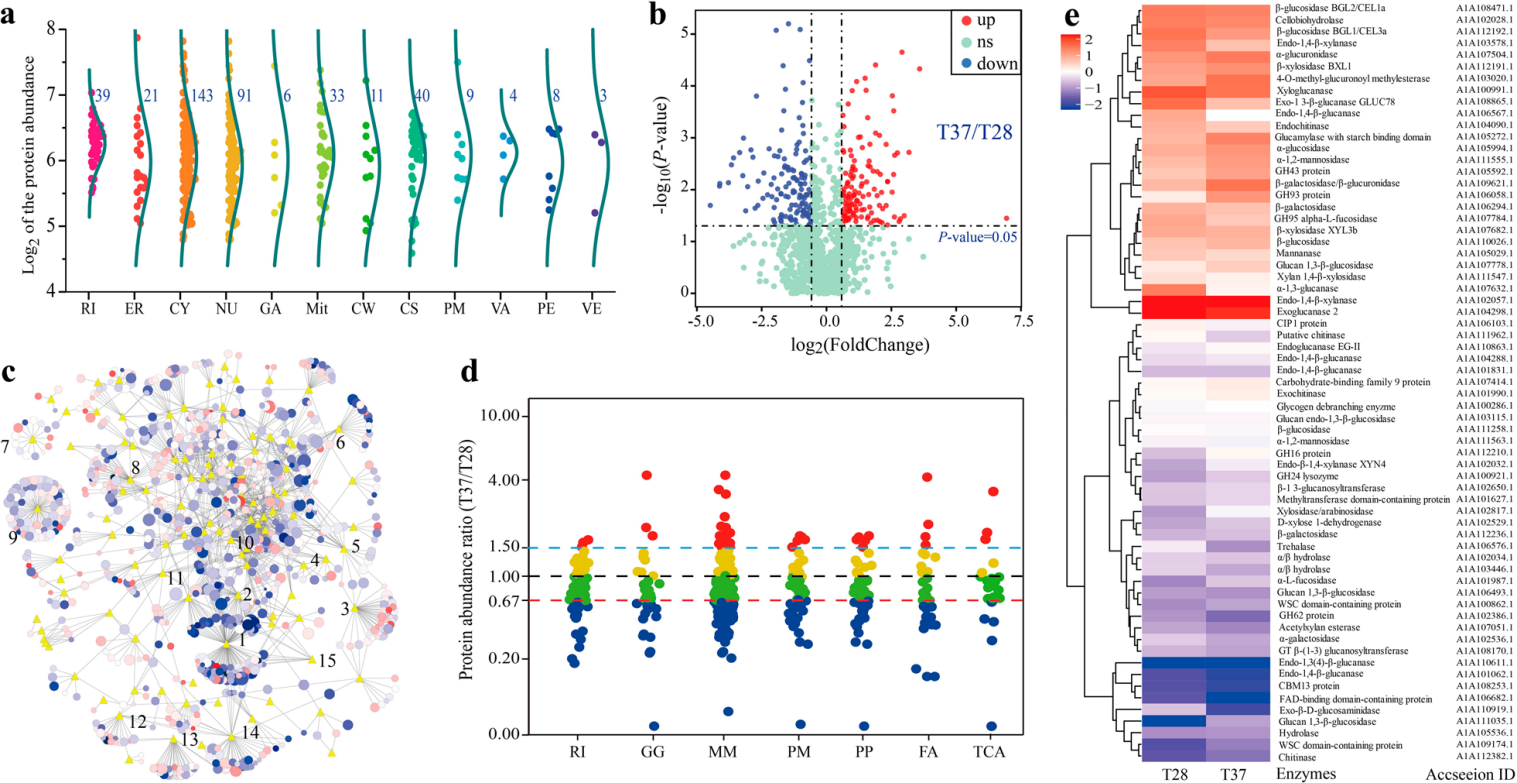
Quantification of the intracellular proteins and carbohydrate-active enzyme (CAZy) family proteins of *T. guizhouense* in different treatments based on SWATH analysis.** a** Location and total abundances for each grouped protein identified by SWATH analysis. RI: ribosome, ER: endoplasmic reticulum, CY: cytoplasm/cytosol, NU: nucleus, GA: Golgi apparatus, Mit: mitochondria, CW: cell wall, CS: cytoskeleton, PM: plasma membrane, VA: vacuole, PE: peroxisome, VE: vesicle. **b** Volcano plot of all the identified proteins with different abundances between T37 and T28. The *P*-value was plotted against the expression fold change of all detected proteins within the different groups. Data points in the lower center area of the plots (cyan circles) display unchanged or proteins with no significant fold change. Data points in the upper quadrants indicate proteins (filled circles) with significant negative (blue circles) and positive (red circles) changes in protein abundance. **c** Protein–protein interaction (PPI) analysis in T37 compared with T28. The nodes are proteins (circles) or KEGG/CAZy categories (yellow diamonds); the edges are protein interactions defined by KEGG or CAZy databases. Protein node sizes indicate the log_10_ of protein expression values. Node colors represent the fold change (red, upregulated; blue, downregulated) of protein expression values between different treatments. Numbers 1 to 15 indicate different pathways. 1: Glycoside hydrolase, 2: starch and sucrose metabolism, 3: oxidative phosphorylation, 4: fatty acid biosynthesis, 5: citrate cycle (TCA cycle), 6: auxiliary activities, 7: proteasome, 8: purine metabolism, 9. Ribosome, 10: Glycolysis/Gluconeogenesis, 11: Carbohydrate esterases, 12: Spliceosome, 13: Transport, 14: Protein processing in the ER, 15: Other glycan degradation. **d** Abundance ratio of the proteins involved in the critical pathway based on SWATH (T37/T28). Nodes represent the fold change (red, upregulated significantly; blue, downregulated significantly; yellow and green, no significant difference) of protein content values between different treatments. RI: ribosome, GG: glycolysis/gluconeogenesis, MM: microbial metabolism in diverse environments, PM: pyruvate metabolism, PP: protein processing in the ER, FA: fatty acid metabolism, TCA: TCA cycle. **e,** Hierarchical cluster analysis of the CAZy family proteins between T28 and T37. Red and blue indicate relatively high and low protein abundance, respectively, and white indicates equal median abundance

To investigate the potential mechanisms underlying the impact of heat stress on the lignocellulases secretion, the proteomes of *T. guizhouense* in T28 and T37 were identified and quantified using the SWATH method. Data quality analysis indicated that the unique protein counts and biological replicates of the samples met the required standards (Additional file 1: Fig. S2). Based on GO (Gene Ontology) analysis, a total number of 1464 differentially expressed proteins were quantified between T28 and T37, mainly involved in binding (36.54%), catalytic activity (52.82%), and structural molecule activity (5.12%) (Additional file 1: Fig. S2). Concurrently, all the identified proteins were classified based on their locations, in which 1226 proteins were annotated as intracellular proteins and 333 proteins were in different detailed intracellular regions, including ribosome (39), ER (21), cytoplasm/cytosol (143), Golgi apparatus (6), mitochondria (33), and nucleus (91) (Fig. 2a). Notably, most intracellular organelles had spindle morphology in the abundance of proteins, and the differences in functional enrichment revealed the different corresponding strategies of *T. guizhouense* under SSF at different temperatures. Besides, with criteria of |log_2_Fold Change|≥ 0.58 and *P*-value < 0.05, 125 and 138 proteins were significantly upregulated and downregulated in T37 compared to T28, respectively (Fig. 2b).

To reveal the relationships between the different proteins that participate in specific metabolic pathways, protein–protein interaction (PPI) analyses were performed between T28 and T37 with Cytoscape software based on the KEGG analysis results (Fig. 2c). Compared to T37, the proteins in T28 clustered in glycoside hydrolase and starch and sucrose metabolism, and most of the proteins that were involved in these pathways were significantly upregulated, which clarified that heat stress could significantly restrict the lignocellulases secretion. Proteins involved in energy metabolism (glycolysis/Gluconeogenesis, TCA cycle, pyruvate metabolism, and fatty acid metabolism) and protein synthesis (ribosome and protein processing in ER) were undoubtedly more abundant and vital when responding to complex conditions [22]. The expression of these proteins, closely related to energy metabolism and protein synthesis, was correspondingly suppressed under heat stress (Fig. 2d). The underrepresentation of energetic core proteins in T37 and the decrease in the specific enzymes involved in energy generation likely contributed to the slow growth and low biomass that were observed. Simultaneously, a lack of protein synthesis and quality control might explain the lower secretion of lignocellulases under heat stress. These results suggested that retaining energy stability and improving the synthesis and processing of proteins are essential steps to promote the production of lignocellulases in *T. guizhouense* under heat stress. Additionally, 238 proteins were annotated as extracellular proteins, mainly including glycoside hydrolases, carbohydrate esterases, auxiliary activities enzymes, proteases, and small secreted cysteine-rich proteins (SSCRPs), in which most proteins were directly involved in lignocellulose degradation and exhibited significant differences. It was worth noting that different hydrolytic enzymes also showed differential expression in T37, which might be due to differences in the transcription levels of genes encoding these enzymes. Additionally, there might be functional similarities among different hydrolytic enzymes, which could also affect protein expression levels (Fig. 2e and Additional file 1: Table S1).

To comprehensively explore the potential regulatory mechanisms, we conducted in-depth PPI analysis of differentially expressed proteins based on the interolog method and the domain interaction-based method [23, 24]. Based on scoring criteria, five proteins were identified as critical proteins responding to the lignocellulases secretion under different temperatures, including NRPS protein (OPB39084), P5CDH (OPB43669), ALDH (KKP01310), ADPRase (OPB41268), and PARP (OPB37503) (Additional file 1: Fig. S3). Surprisingly, it was found that ALDH, PARP, and ADPRase were all involved in the metabolism of NAD^+^ and ATP, and the latter two were present in the poly ADP-ribosylation pathway (Additional file 1: Fig. S3). This conveyed a message: whether NAD^+^ and ATP metabolism were key factors linking heat stress to the lignocellulases secretion in *T. guizhouense*. Although the functions of homologous protein of PARP and ADPRase involved in abiotic stress have been previously addressed in plant [25, 26], the internal regulatory mechanism of growth and lignocellulases secretion in filamentous fungi under heat stress has not been stated clearly. Therefore, we attempted to explore the roles of *Tg*PARP (*Tgparp*) and *Tg*ADPRase (*Tgadprase*) of *T. guizhouense* in responding to heat stress and lignocellulases secretion.

### Gene Tgparp and Tgadprase respond to lignocellulases secretion of T. guizhouense under heat stress

**Fig. 3:**
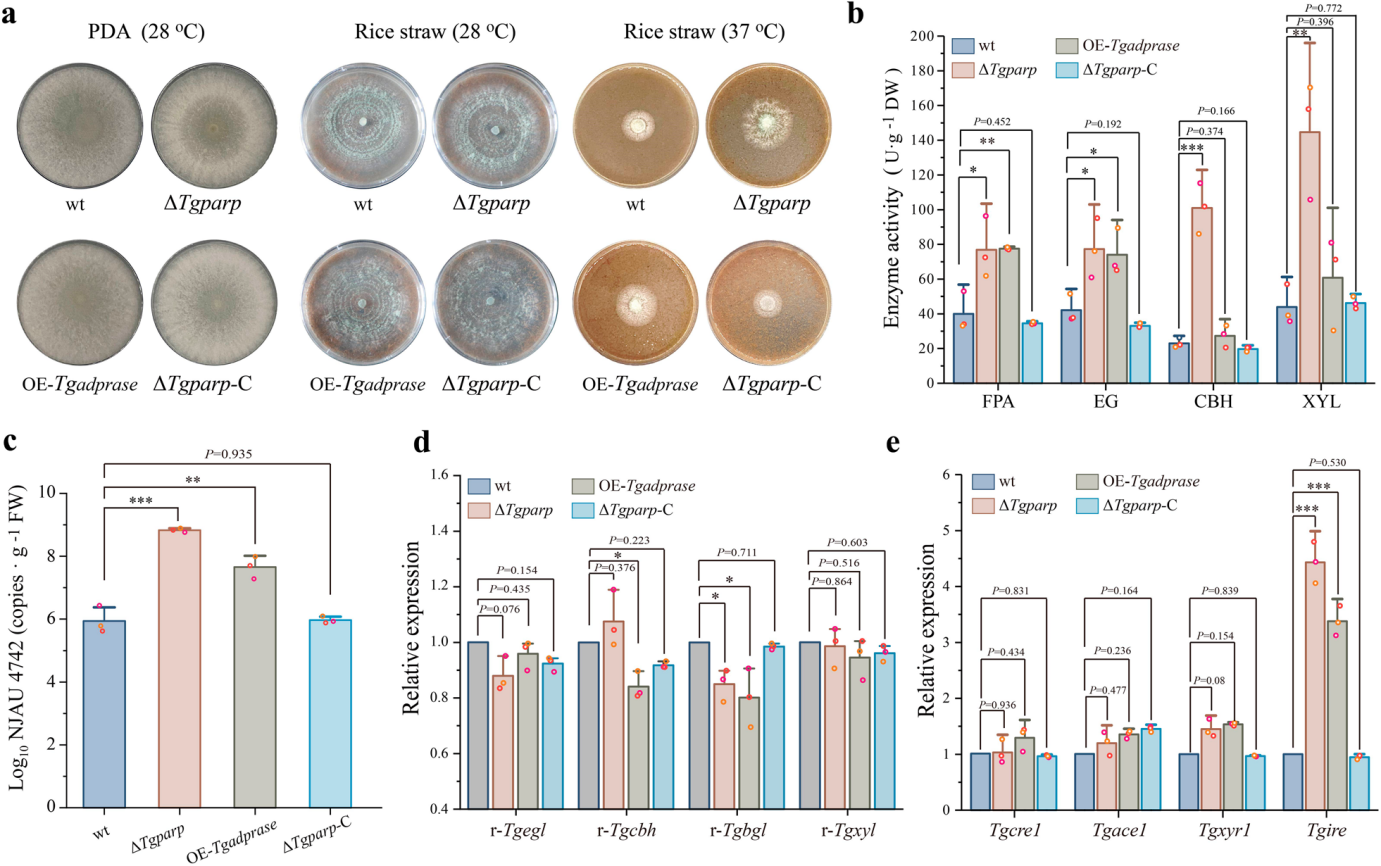
Promotion of lignocellulose utilization efficiency of *T. guizhouense* by deleting *Tgparp* or overexpressing *Tgadprase* under heat stress. **a** Growth comparison of the wt and mutants inoculated on PDA medium for 72 h at 28 °C and rice straw medium for 48 h at 28 °C and 37 °C. **b** Hydrolase activities, including FPA, EG, CBH, and XYL, of wt and mutants using rice straw as the sole carbon source under heat stress. **c,** Biomass of *T. guizhouense* by quantifying the copies of a 300 bp fragment from *T. guizhouense* in different treatments under heat stress (values are shown as the log_10_ conversion of the fragment copies·g^−1^ FW rice straw-induced cultures). **d, e** Transcriptional levels of some representative lignocellulase genes and regulatory factors in different treatments based on SWATH analysis. The representative lignocellulase genes included r-*Tgegl* (endo-1,4-β-glucanase, OPB37031), r-*Tgcbh* (cellobiohydrolase, OPB45635), r-*Tgbgl* (β-glucosidase, KKP01743), and r-*Tgxyl* (endo-1,4-β-xylanase, OPB45659). The regulatory factors included *Tgace1* (OPB46882.1), *Tgcre1* (OPB38360.1), *Tgxyr1* (OPB38038.1), and *Tgire* (OPB43384.1). Data were calculated from three biological replicates, and error bars represent ± SDs. * *P* < 0.05, ** *P* < 0.01, *** *P* < 0.001. A *P-*value < 0.05 was regarded as statistically significant

Based on the above speculation, we constructed Δ*Tgparp* (deletion of gene *Tgparp*) and OE-*Tgadprase* (overexpression of gene *Tgadprase*), and the mutants were confirmed by the southern blot and quantitative RT-PCR analysis (Additional file 1: Figs. S4–S5), respectively. Both mutants exhibited no difference in vegetative growth compared to wild type (wt), while showing lusher biomass compared to wt using rice straw as the sole carbon source under heat stress. However, the growth of Δ*Tgparp* complemented strain (Δ*Tgparp*-C) was restored to wt (Fig. 3a). Moreover, the carbon utilization characterizations of wt, Δ*Tgparp*, and OE-*Tgadprase* were investigated by the Biolog FF microplates analysis, which indicated that OE-*Tgadprase* increased the utilization capacity of some carbon source substrates, while Δ*Tgparp* exhibited a similar efficiency as that of wt (Additional file 1: Fig. S6). This would exclude the effect of the metabolic discrepancy in Δ*Tgparp* on the carbon transformation of the primary carbon source. Meanwhile, the lignocellulases activity assays revealed that, in comparison to wt, FPA (filter paper activity), EG (endoglucanase), CBH (cellobiohydrolase), and XYL (xylanase) activities were increased by 1.93-, 1.84-, 4.39-, and 3.30-fold in Δ*Tgparp*, respectively, and they were also increased by 1.94-,1.76-,1.18-, and 1.39-fold in OE-*Tgadprase*, respectively (Fig. 3b). Consistent with the enzyme activities, the biomass of Δ*Tgparp* and OE-*Tgadprase* achieved copy numbers of 1 × 10^8.83^ and 1 × 10^7.66^ on the fourth day during SSF under heat stress, respectively, which were both greater than those of wt (1 × 10^5.95^) and Δ*Tgparp*-C (1 × 10^5.97^) (Fig. 3c).

To elucidate the molecular mechanisms underlying the mediation of lignocellulases secretion by genes *Tgparp* and *Tgadprase*, a targeted subset of lignocellulase genes and regulatory factors (*Tgcre*1, *Tgace*1, *Tgxyr*1, and *Tgire*) were initially quantified after 72 h of cultivation using straw as the sole carbon source, aiming to probe potential transcriptional regulatory processes. Remarkably, the expression levels of lignocellulase genes and regulatory factors (*Tgcre*1, *Tgace*1, and *Tgxyr*1) exhibited no significant distinctions among the Δ*Tgparp*, OE-*Tgadprase*, and wt (Fig. 3d and Additional file 1: Fig. S7). However, a pronounced upregulation of *Tgire* was observed, with a 4.44-fold increase in Δ*Tgparp* and a 3.12-fold increase in OE-*Tgadprase*, respectively (Fig. 3e). Given the well-established link between severe ER stress induced by heat stress and the role of IRE in orchestrating the UPR [27], the regulatory mechanism of *Tgire* assumes paramount importance. Thus, we hypothesized that genes *Tgparp* and *Tgadprase* potentially modulated lignocellulases secretion by influencing the transcriptional regulation of *Tgire*, thereby bolstering the ER stress under heat stress.

### The functions of Tgparp in activating mitochondrial activity under heat stress

The functional genes *parp* and *adprase* are mainly involved in protein poly(ADP-ribosyl)ation (PARylation), and the recycling of ADP-ribose may represent an important pathway to re-establish ATP that can be utilized by the cell [28, 29]. Besides, it is well documented that PARP inhibitors could protect against oxidative stress-induced cell death and prevent the oxidative stress-induced depolarization of MMP [30]. In light of the functions above of PARP and the superior lignocellulases secretion capacity observed in Δ*Tgparp* compared to OE-*Tgadprase* under heat stress, strain Δ*Tgparp* was chosen as the experimental material to scrutinize the intricate relationship between gene *Tgparp*, ATP content, and MMP.

Firstly, ATP levels were quantified in both wt and Δ*Tgparp* as the temperature progressively increased from 28 °C to 37 °C. The outcomes revealed a gradual elevation in ATP content within the Δ*Tgparp* compared to wt when the temperature reached 34 °C (Additional file 1: Fig. S8). This result might offer insights into the substantial growth inhibition experienced by *T.guizhouense* beyond 34 °C (Additional file 1: Fig. S1), suggesting a potential correlation between gene *Tgparp* activation and ATP consumption in response to heightened heat stress. To further elucidate the compensatory role of ATP under heat stress, exogenous ATP-Na_2_ was introduced into the culture medium at varying concentrations, suggesting the addition of 500–1000 μM ATP-Na_2_ significantly ameliorated the growth of *T.guizhouense* (Additional file 1: Fig. S9).

**Fig. 4:**
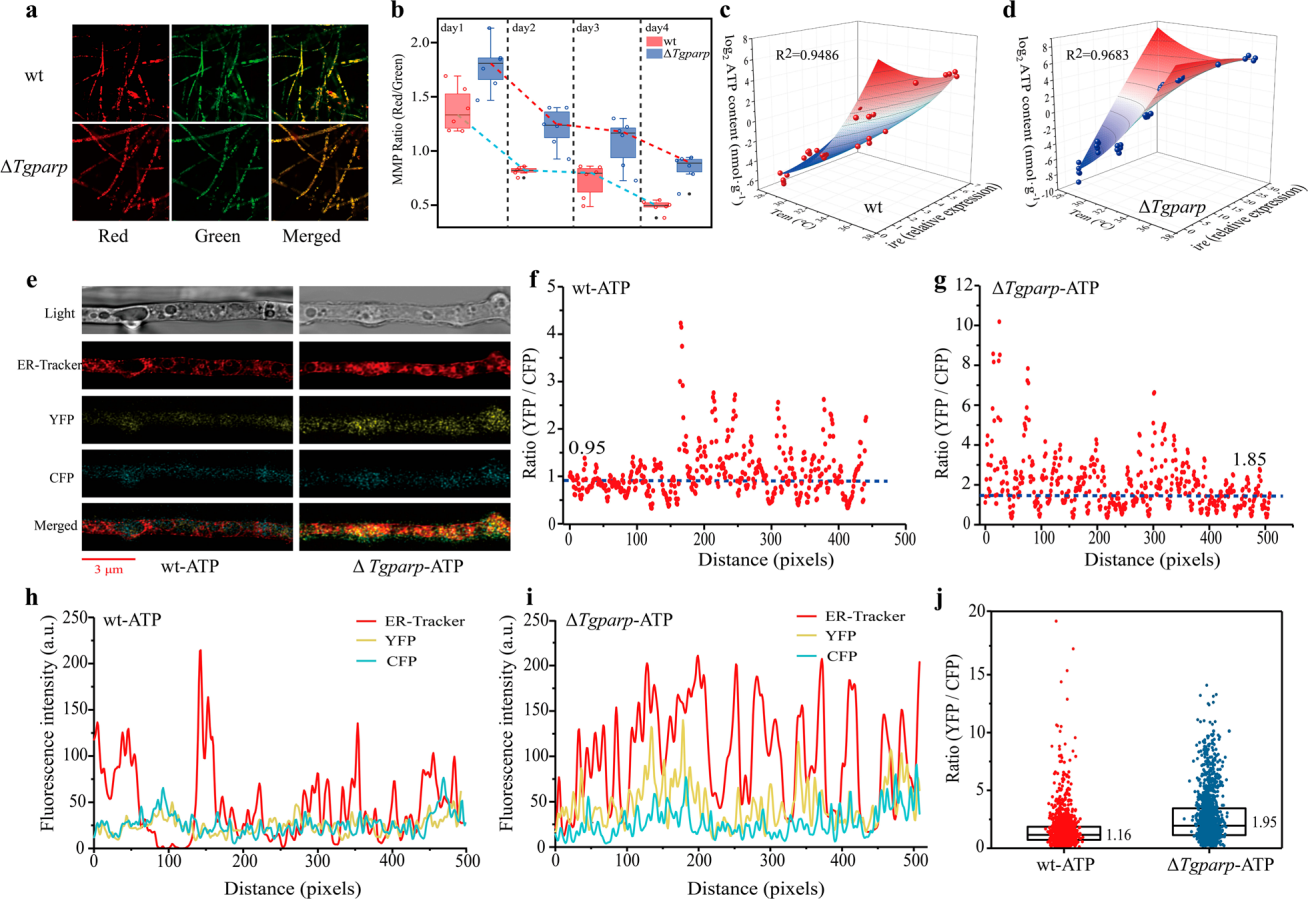
Deleting *Tgparp* could enhance the protective effect of MMP, thereby allocating more ATP to the ER of *T. guizhouense* under heat stress.** a** Hyphae characterization of wt and Δ*Tgparp* after staining with JC-10; **b** Level of mitochondrial membrane potential (MMP) in wt and Δ*Tgparp* under heat stress, determined by evaluating the ratio of red to green fluorescence intensity. The box plot shows the median, lower and upper quartiles, and minimum and maximum the ratio values for each sample. **C, d** Relationship between ATP content and *ire* expression level with a gradual increase in temperature (from 28 °C to 37 °C) between wt and Δ*Tgparp*. X-axis: temperature; Y-axis: the expression of *ire* relative to wt at 28 °C; Z-axis: the log_2_ of ATP content (nmol·g^−1^). **e** Confocal imaging analysis of hyphae from wt-ATP (left) and Δ*Tgparp*-ATP (right) strains after staining with ER-Tracker™ Red, and bar = 3 μm. **f, g** ATP levels plotted for single pixels of the mycelium expressing the ATP sensor in wt-ATP and Δ*Tgparp*-ATP. The ratios of the YFP/CFP emission intensities in each region with the ER pixels in wt-ATP (left) and Δ*Tgparp*-ATP (right). **h, i** Fluorescence intensity of the three channels (ER‑Tracker, YFP, and CFP) detected in wt-ATP (left) and Δ*Tgparp*-ATP (right). **j** Ratios of the YFP/CFP emission intensities from more mycelial samples between wt-ATP and Δ*Tgparp*-ATP, and the median was utilized to assess ATP level

MMP provides the driving force for ATP synthesis in mitochondria, and MMP stability is considered a requisite for energy homeostasis and normal cell functioning [31]. Here, monitoring of MMP was conducted using a membrane potential-dependent fluorescence dye, which can form red fluorescent aggregates in healthy mitochondria (indicative of higher MMP) and predominantly generates green fluorescence in unhealthy mitochondria (indicative of lower MMP) [32]. The results indicated that Δ*Tgparp* exhibited a diminished intensity in green fluorescence, while the red fluorescence remained relatively stable compared to wt (Fig. 4a). Moreover, a precise assessment of the ratio between red and green fluorescence intensities was conducted across successive fermentation cycles. The outcomes revealed a declining trend in this ratio for both wt and Δ*Tgparp*, likely attributable to sustained reductions in MMP induced by temperature and nutrient stress. However, notably, Δ*Tgparp* consistently demonstrates a higher ratio compared to wt (Fig. 4b). These findings indicated that gene *Tgparp* deletion might enhance the protective role of MMP, thereby mitigating mitochondrial damage under heat stress. Previous studies have shown that intracellular ROS levels are also important factors influencing MMP, especially when ROS is induced in mitochondria and subsequently leads to significant oxidation of the mitochondrial membrane, which may also directly affect the level of cellular ATP and thus affect MMP [33, 34]. Thus, under heat stress, the loss of *Tgparp* might reduce the excessive accumulation of intracellular ROS, thereby maintaining a more stable MMP. Additionally, constructing a multiple linear regression model to elucidate the intrinsic connection between *Tgire* transcription level and ATP content in both Δ*Tgparp* and wt, as the temperature gradually increased. Surprisingly, the models built using both strains showed high reliability, and the R^2^ value of the model reached 0.9486 in wt and even higher at 0.9683 in Δ*Tgparp*, indicating a significant positive correlation between *Tgire* transcription level and ATP content, especially under heat stress (Fig. 4c, d).

In addition, single-gene mutant Δ*Tgire* and double-gene mutant Δ*Tgire*Δ*Tgparp* were also constructed. We observed a significant biomass reduction in Δ*Tgire*, and the deletion of *Tgparp* based on the Δ*Tgire* did not improve its compromised state (Additional file 1: Fig. S4). These findings highlighted the crucial role of *Tgire* in alleviating heat stress and the positive correlation between ATP content and *Tgire* expression levels.

### In situ quantitative analysis of ATP gathered in the ER lumen

Proper protein folding in the ER is vital and believed to be an evolutionarily conserved mechanism in eukaryotes. When misfolded proteins accumulate in the ER lumen, IRE can expand the folding capacity and increase the degradation of misfolded proteins in the ER [35]. Protein folding, refolding, and degradation are energy-consuming processes, that can be promoted through kinetic partitioning in the ATP-dependent mechanism of chaperone action, and ATP-triggered release allows folding to proceed [36]. Furthermore, two seemingly disparate stressors, heat stress and nutrient deprivation, might trigger the loss of intracellular ATP [37]. Thus, two mutants (wt-ATP and Δ*Tgparp*-ATP) for tracking the intracellular ATP content were generated by inserting the plasmid AT1.03, in which a genetically encoded FRET-based ATP sensor with a higher 527:475 nm emission ratio was indicative of higher ATP concentration [38].

The mycelial vegetative growth of these two mutants exhibited a similar trend to that of wt, while a significant increase in biomass was observed in Δ*Tgparp*-ATP compared to wt-ATP (Additional file 1: Fig. S10). Then, ATP content was quantified in the ER after staining by the ER-Tracker™ Red (Fig. 4e), and the emission ratios of YFP / CFP in each region with ER pixels were measured. The different fluorescence intensities of each pixel are displayed in Fig. 4i, j, and the ratios were calculated. Notably, compared to wt-ATP (0.95), a higher YFP / CFP ratio was detected in Δ*Tgparp*-ATP (1.85) (Fig. 4f, g). Extensive microscopic sample analysis results confirmed the same trend, and there was an approximately two-fold increase in Δ*Tgparp*-ATP relative to wt-ATP (Fig. 4h). These findings suggested that Δ*Tgparp* accumulated higher levels of ATP in the ER, potentially contributing to protein folding and refolding, as well as the degradation of misfolded proteins, to minimize the aggregation of misfolded proteins in the ER.

### Monitoring the allocation and secretion of different lignocellulases

**Fig. 5:**
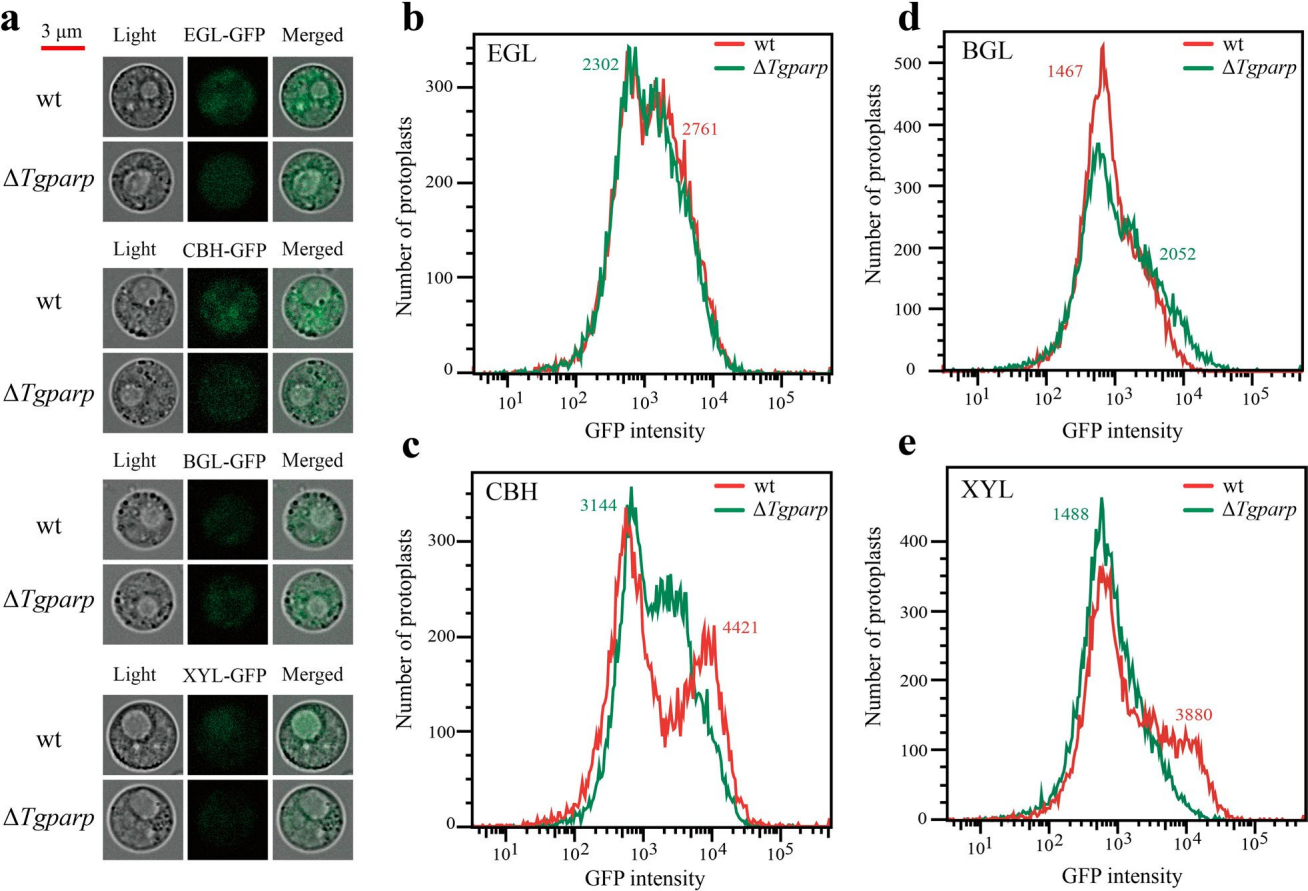
Analysis of lignocellulases-GFP in the protoplasts between wt and Δ*Tgparp*. **a** Confocal images of protoplasts from different lignocellulases-GFP fusion strains, and bar = 3 μm. **b–e** Fluorescence intensities of protoplasts from different lignocellulases-GFP strains analyzed using flow cytometry. The MdFI of different lignocellulases-GFP in wt and Δ*Tgparp* is shown in **b** (EGL), **c** (CBH), **d** (BGL), and **e** (XYL)

**Fig. 6:**
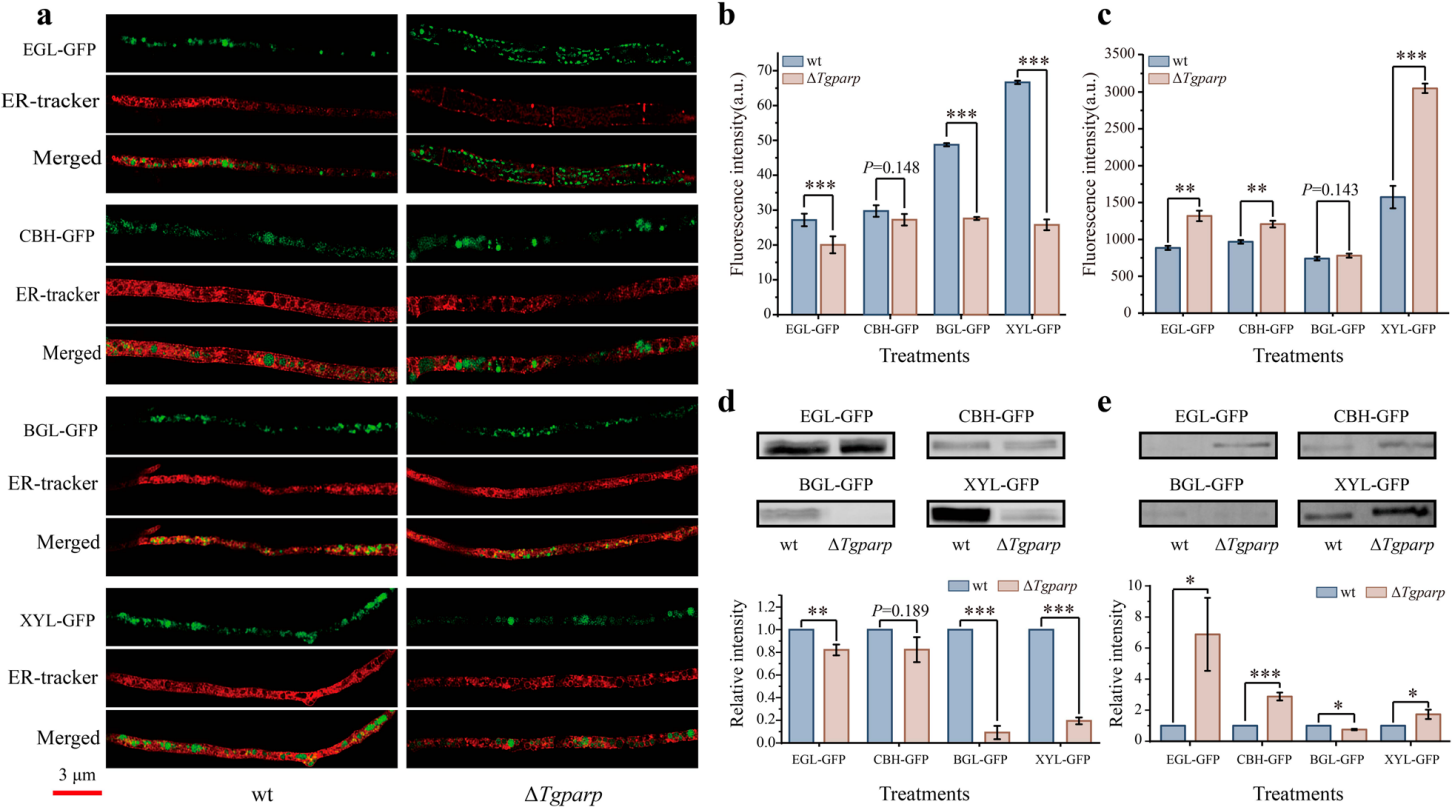
Localization analysis of lignocellulases-GFP in the ER and extracellular region between wt and Δ*Tgparp*.** a** Confocal images of hyphae from the lignocellulases-GFP fusion strains after staining with ER-Tracker™ Red to track lignocellulases localization in the ER between wt and Δ*Tgparp*. **b** Average intensity of lignocellulases-GFP fluorescence in the ER of wt and Δ*Tgparp* after staining hyphae with ER-Tracker™ as markers of ER compartment. Data were calculated from all valid pixels in the ER, and error bars represent ± SEs. **c** Fluorescence intensities of fermentation supernatant when SSF of different lignocellulase-GFP fusion strains was carried out for 4 days. Data were calculated from three biological replicates. Error bars represent ± SDs. **d–e** Western blot analysis of lignocellulase-GFPs in the ER (left) and fermentation supernatants (right), and the intensity of Western blotting bands was compared between wt and Δ*Tgparp*. Three independent biological experiments were performed and yielded similar results in each independent biological experiment. Error bars represent ± SDs. * *P* < 0.05, ** *P* < 0.01, *** *P* < 0.001. A *P-*value < 0.05 was regarded as statistically significant

A protein quality control system ensures that the correctly folded proteins are delivered to subsequent cellular compartments and regulates the folding status of proteins to prevent the aggregation of misfolded proteins in the ER by either refolding or degrading aggregation-prone species [39]. Aging or stress conditions could lead to the exhaustion of this protective system, causing the generation of protein aggregates [40]. To confirm that higher ATP in the ER could reduce the aggregation of the misfolded proteins, we reasoned that more lignocellulases should be secreted outside the fungal cell instead of gathering in the intracellular region, especially in the ER. Consequently, different GFP-tagged lignocellulases were generated to track their distribution in wt and Δ*Tgparp*. Initially, flow cytometry was employed for in situ analysis of intracellular fluorescence intensity to characterize the aggregation of lignocellulases in cellular regions (Fig. 5a). Notably, the fluorescence intensity of all events exhibited an approximately normal distribution (Additional file 1: Fig. S11). The median fluorescence intensity (MdFI) of these events was calculated to quantify the expression levels of distinct cellulases within fungal cells [41]. The results indicated that, except for BGL, the intracellular accumulation levels of EGL, CBH, and XYL in Δ*Tgparp* were lower than in wt (Fig. 5b–e). To further elucidate the aggregation level of different lignocellulases in the ER, the fluorescence intensity of different lignocellulases-GFP was detected after staining by the ER-Tracker™ Red (Fig. 6a). The average intensity of GFP fluorescence was calculated to measure the expression levels of lignocellulases-GFP in the ER. As shown in Fig. 6b, the levels of lignocellulases-GFP distributed in ER were significantly higher in wt compared to Δ*Tgparp*, including EGL-GFP, CBH-GFP, BGL-GFP, and XYL-GFP. Simultaneously, the lignocellulases secretion was also detected by determining the fluorescence intensity of the supernatants extracted from the SSF. As expected, the fluorescence intensity of EGL-GFP, CBH-GFP, and XYL-GFP of Δ*Tgparp* was significantly higher than that in wt (Fig. 6c). Finally, ER proteins and secreted proteins of *T. guizhouense* were extracted and collected from recombinant lignocellulase-GFP strains during SSF. Through GFP immunoblot analysis, the results revealed a significant enrichment of EGL-GFP, BGL-GFP, and XYL-GFP in the ER of wt compared to Δ*Tgparp* (Fig. 6d). However, Δ*Tgparp* exhibited a heightened extracellular secretion capacity for EGL-GFP, CBH-GFP, and XYL-GFP relative to wt (Fig. 6e). In conclusion, these findings suggested that increased ATP assembly in the ER promoted the functionality of *Tg*IRE, minimizing the lignocellulases aggregation in the ER, thereby enhancing the lignocellulases secretion of *T.guizhouense* under heat stress.

## Discussion

All biological systems generally operate in narrow temperature ranges specific to particular species. Changes in temperature out of the optimal range may cause abnormalities in nearly all biological processes. However, the low growth efficiency of soil fungi at high temperatures can provide feedback. As external environmental temperatures fluctuate, fungal cells have developed complex systems to recognize the state of their environment and activate defense mechanisms to resist environmental stress [42]. It was widely accepted that keeping energy homeostasis was vital to conquer various stresses for fungal cells [43]. Besides, it has been reported that the folding of many secretory proteins could be inhibited by depleting cellular ATP levels and then strengthened by increasing ATP levels [44–46]. This work revealed that lignocellulases secretion might be broadly modulated by regulating cellular ATP content under heat stress. This ATP regulation could reduce the negative effect of heat stress on MMP through building energy homeostasis, which could relieve the ER stress when sufficient ATP is gathered in the ER. Besides, we proposed that ER stress might be mitigated through an energy homeostasis state by deleting *Tgparp*, which could contribute to the proper folding of various lignocellulases and avoid the aggregation in the ER to promote their secretion into the extracellular space using rice straw as the sole carbon source under heat stress.

Inevitably, DNA repair through the base excision repair pathway is always mentioned when studying the functions of PARP family members in different species [47, 48]. In *P. Anserina*, the *PaParp* gene seemed necessary for the proliferation and metabolism, and extensively attempted to delete the gene via homologous recombination had never been successful [49]. This result was in concordance with that in mice, in which knockout of *parp*-1 and *parp*-2 separately was found to be lethal [50], and the same results were also obtained in *A. nidulans* [51]. The deletion of the *Npo* gene encoding the ortholog with *parp* in *Neurospora crassa* had succeeded, and the researchers found that the *Npo* gene was optional and not required for heterochromatin formation [52]. These results clearly indicated that the PARP family proteins had diverse functions and exhibited a certain degree of specificity across different species. To avoid the loss of some unknown functions owing to the complete absence of *Tgparp*, whose suppressor mutants (*Tgparp*-RNAi) were generated, and various relevant enzyme activities indicated that more efficient lignocellulases secretion was detected than that in wt (Additional file 1: Fig. S12). Besides, the quantitative results of transcription levels for three DNA repair genes (*Tgalkb*, *Tgrad50*, and *Tgrhp54*) revealed significant upregulation of the *Tgalkb* gene in the Δ*Tg*parp compared to wt, whereas this upregulation was not observed in *Tgparp*-RNAi (Additional file 1: Fig. S12). These results further suggested that, similar to other species, the DNA repair function of the *Tgparp* gene existed in *T. guizhouense* but was not essential. Therefore, appropriately inhibiting the *Tgparp* transcription level might be crucial for establishing a balance between DNA repair and ATP consumption, thereby promoting cellular resistance to heat stress and maintaining the lignocellulases secretion.

**Fig. 7:**
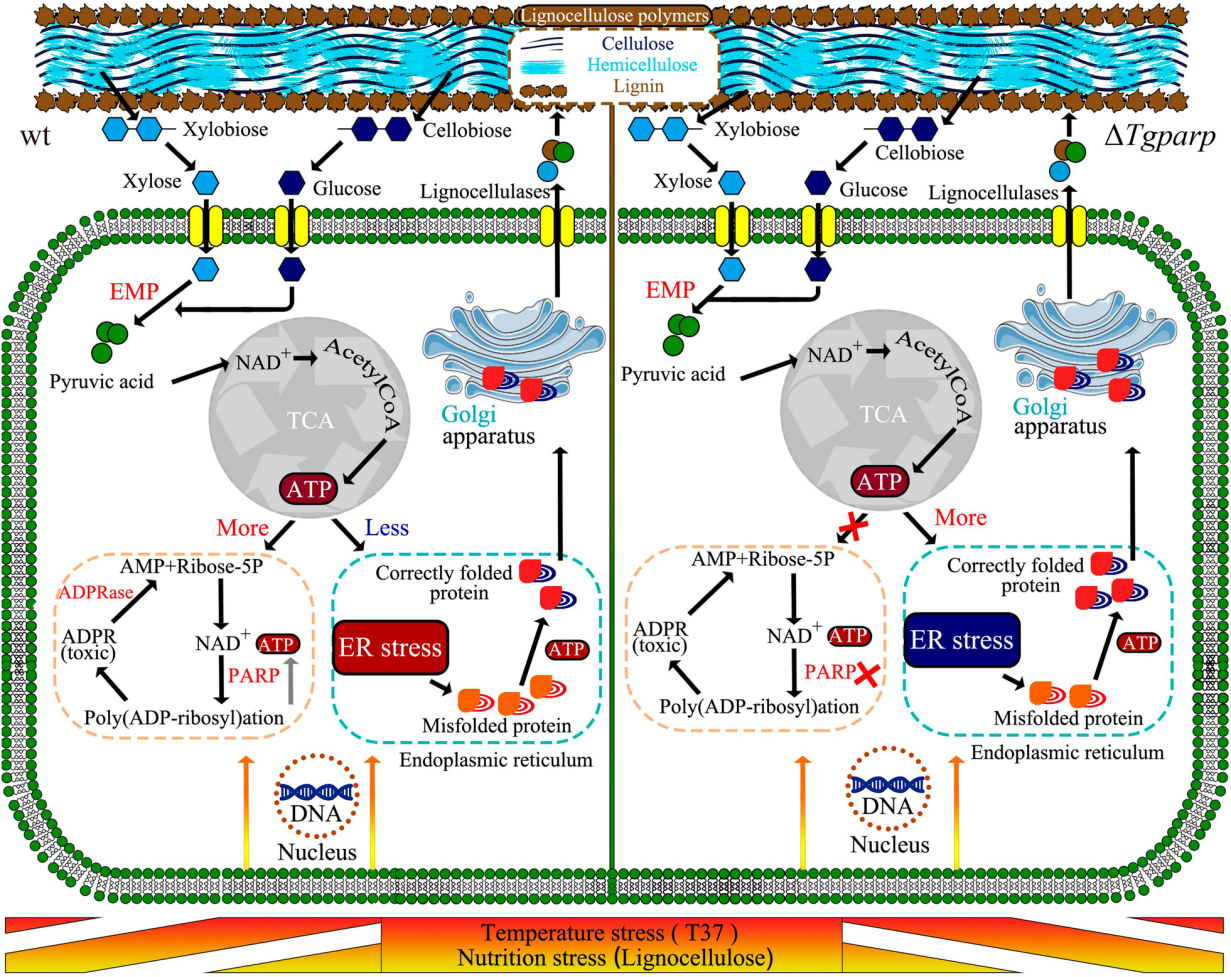
Proposed model for enhancing the lignocellulases secretion in *T. guizhouense* under heat stress by deleting the gene *Tgparp*. ER stress and gene *Tgparp* expression could be activated by heat stress and nutrition stress. Deletion of *Tgparp* maintains energy homeostasis, allocating more ATP to the ER for protein folding or refolding. This prevents excessive protein aggregation in the ER, thereby promoting the lignocellulases secretion of *T. guizhouense* under heat stress

Usually, PARP activity depends on NAD^+^ levels, and a large amount of energy consumption accompanies the generation of NAD^+^ [53]. In the course of this investigation, two additional proteins identified in the protein PPI analysis, namely, P5CDH, and ALDH, were found to exhibit a preference for participating in NAD^+^ metabolism. P5CDH and ALDH are two nicotinamide adenine dinucleotide (phosphate) (NAD(P))-dependent enzymes involved in detoxification, biosynthesis, antioxidant functions, and regulatory mechanisms. These biological processes are typically accompanied by the conversion from NAD^+^ to NADH [54]. Importantly, these proteins exhibited significant downregulation in T37 compared to T28, potentially preventing excessive NAD^+^ depletion and redirecting energy consumption toward the resynthesis of NAD^+^. This hypothesis was further substantiated by in situ detection of NAD^+^, employing constructed sensors for in situ tracking of NAD^+^ (mCherry-cpYFP and mCherry-FiNad). As the control, the mCherry-cpYFP fluorescence ratio did not significantly change in wt (1.22) and Δ*Tgparp* (1.32). Notably, compared to the mCherry-FiNad fluorescence ratio of wt (2.41), a lower ratio (1.75) was detected in Δ*Tgparp*. Normalized to the control, the NAD^+^ ratio decreased by approximately 1.5-fold in Δ*Tgparp* compared to wt (Additional file 1: Fig. S13 and S14). Therefore, we boldly speculated that more NAD^+^ was synthesized to be the substrate for *Tg*PARP activation and was accompanied by tremendous ATP consumption. In fact, this process was a “waste” of ATP in *T. guizhouense* under heat stress. Furthermore, Persson et al. suggested that slowing some cellular processes could conserve energy under stress conditions [37]. Thus, a large amount of energy expenditure caused by the overactivation of *Tgparp* was more pernicious compared with the replaceable function of DNA repair under heat stress. On the other hand, communication between mitochondria and the ER has been reported to be primarily associated with the transfer of ions and lipids, signal transduction, and membrane dynamics [55]. Here, we propose an alternative connection: avoiding excessive consumption of NAD^+^ and ATP by inhibiting *Tgparp* activation can lead to healthier mitochondria. Stable mitochondria can establish energy homeostasis, allocating more ATP in the ER to reduce protein overaccumulation, thereby enhancing the lignocellulases secretion of *T. guizhouense* under heat stress (Fig. 7).

## Conclusion

This study provided insights into the significant impact of heat stress on the lignocellulases secretion in *T. guizhouense* and elucidated the potential mechanisms underlying this process. *Tg*PARP was activated under heat stress, leading to accelerated depletion of intracellular ATP. Deletion of gene *Tgparp* led to a significant increase in intracellular ATP levels and maintained a higher MMP. Simultaneously, knocking out *Tgparp* promoted the transcription level of an ER stress response factor (*Tgire*), and its transcription level significantly correlated with intracellular ATP levels. Further investigations indicated that the absence of *Tgparp* regulated the intracellular distribution of ATP, leading to increased ATP accumulation in the ER, and enhancing protein folding and repair. This alleviated the accumulation of lignocellulases in the ER and enhanced their extracellular secretion. In conclusion, this study elucidated a novel pathway and mechanism for enhancing lignocellulose utilization of filamentous fungi under heat stress.

## Materials and methods

### Strains and culture conditions

*Trichoderma guizhouense* NJAU4742 was used as the wild-type strain for all experiments, and its genome sequence was already published in the NCBI database (accession No. LVVK00000000.1) and *T. guizhouense* genome website (https://bioinfo.njau.edu.cn/tgn4742/). To assess the lignocellulose utilization capacity and lignocellulases secretion, 2% (w/v) rice straw powder was added to the Mandels' salt solution without organic components and thoroughly mixed to generate straw agar plates and solid fermentation medium. Fresh spores of *T. guizhouense* were collected from PDA plates and filtered through four layers of cheesecloth. The spore concentration was quantified using a hemocytometer and adjusted to 1.0 × 10^7^ spores·mL^−1^. The strains with equal biomass were transferred to the rice straw agar medium to observe the growth and biomass. A volume of 1 mL of *T. guizhouense* spores was inoculated for SSF, and all treatments were performed at 28 °C and 37 °C for seven days, with samples collected between day 2 and day 7. Each sampling point had three biological replicates.

### Assays of enzyme activities and CO2 evolution rate

The endoglucanase activity and filter paper activity (FPA) were measured using CMC-Na (Sigma, USA) and filter paper (Whatman No.1) as substrates, following the method described by Xue et al. [56]. Xylanase activity was assayed with oat spelts xylan (Sigma, USA) as the substrates [57]. The assays for these three enzymes were conducted at 50 °C for 10 min in a 0.1 M acetate buffer (pH 4.8). Subsequently, the released reducing sugars were measured using the DNS method [58]. The cellobiohydrolase activity was determined in 0.1 M acetate buffer at 50 °C for 30 min with pNPC (Sigma, USA) as substrates according to Liu et al. [59]. One enzyme activity unit was defined as the number of enzymes required to liberate one μmol glucose or pNP per minute under the assayed conditions. The respiratory rate of *T. guizhouense* under SSF was evaluated by tracking the release of CO_2_. Briefly, the triangular flasks for SSF were sealed with a parafilm for 2 h before sampling, and then 20 mL of gas was extracted with a syringe. The collected gas was injected into the gas sampling bag (E-SWITCH, China), and the contents of CO_2_ were determined by gas chromatography (Agilent 7890A) equipped with a Porapak Q column and a flame ionization detector (FID) according to Zhang et al. [60].

### Protein extraction and SWATH analysis

Protein extraction was performed after seven days' SSF as follows: 150 mL deionized ddH_2_O was added into the triangular flasks and then shaken at 180 rpm for 1 h, and the supernatants were collected by centrifugation at 12,000 rpm for 10 min and filtration through a 0.22 μm millipore filter membrane (Merck Millipore, German) for removing the impurities. The precipitates of the former step were used to extract the mycelial intracellular proteins using the NoviPure® Soil Protein Extraction Kit (MOBIO, USA) according to the manufacturer's protocol. The extracellular and intracellular proteins, both extracted from the same amount of fermentation substrates (mg·g^−1^ DW, protein/substrates), were directly mixed and concentrated by lyophilization (Powerdry LL3000, HETO, Denmark). The protein samples were stored at −80 °C for the subsequent experiment.

The SWATH (sequential window acquisition of all theoretical spectra) analysis of the proteomes was commissioned by a specialized company (GeneCreate, Wuhan, China). The protocols were described briefly as follows: 100 μg of proteins in different treatments was digested by trypsin (Promega, USA) at 37 °C overnight and then separated by the StrataX C18 pillar (Phenomenex, Torrance, CA, USA) and dried using a vacuum concentration meter. LC–ESI–MS/MS analysis was performed on an ABSCIEX nanoLC-MS/MS (Triple TOF 5600 plus) system. Data-dependent acquisition (DDA) was followed by SWATH acquisition, where all samples were mixed and detected in DDA, and the resulting data were used as a library for the analysis of each sample by SWATH. Spectral library generation and SWATH data processing were performed using skyline version 3.5 software, complying with the rules raised by Röst HL et al. [61]. Following the above instructions, each ion's XIC was automatically extracted with a retention time width of 5 min, and the area under the XIC curve (AUC) for individual ions was calculated. Fragment ions' areas that belong to one peptide were summed to get a peptide's abundance, and a summed abundance of peptides for a given protein was conducted to get the protein's abundance. To eliminate the random errors and sample bias, we normalized all the data among samples using a median normalization method [62]. Skyline employed the mProphet algorithm toward each extracted peak to assess the data confidence and control the false discovery rate.

### Generation of *T. guizhouense* targeted gene deletion and overexpression mutants

Gene deletion and overexpression mutants were obtained through a gene replacement strategy using the polyethylene glycol (PEG)-mediated protoplast transformation procedure as described in Zhang et al. [63], with a hygromycin resistance cassette (*HygB*) in wt strain, or a *Tgura3* compensation on *Tgura3*-deleted strain (Δ*Tgura3*) to screen mutants without uridine. The detailed protocols were described in Additional file 1: Fig. S4, and the transformants were screened by PCR with a Phire® Plant Direct PCR Kit (Thermo Scientific, USA) and subjected to three rounds of single‐spore purification. For reverse complementation of Δ*Tgparp*, a copy of the gene with its promoter and terminator region from the wt strain fused with the *HygB* gene at the C-terminus was amplified and inserted into Δ*Tgparp* that was constructed by introducing *Tgura3*. The primers involved in the vector construction process are shown in Additional file 1: Table S2.

### Quantitative RT-PCR and biomass assays of *T. guizhouense*

For mycelial qRT-PCR, total RNA extraction was performed using the RNeasy® Plant Mini Kit (Qiagen, Germany), and cDNA synthesis was completed using the PrimeScript RT Reagent Kit (RR036A, Takara, Dalian, China) according to the manufacturer's instructions. The qRT-PCR was performed using SYBR Premix Ex Taq II (RR820A, Takara, Dalian, China) and the CFX Connect Real-Time System (Bio-Rad, Hercules, USA).

For biomass assay, four-day-old *T. guizhouense* mycelia growing at 37 °C under SSF were collected and mixed evenly, and the total DNA was extracted with the EZNATM Soil DNA kit (Omega Bio-Tek, Inc., Norcross, GA, USA). The number of *T. guizhouense* copies was determined with SYBR Premix Ex Taq II (RR820A, Takara, Dalian, China), and the amplification of the fragment using the extracted DNA as a model was performed on a CFX Connect Real-Time System (Bio-Rad, Hercules, USA).

### ATP content and MMP assays

ATP content was determined using the ATP Content Kit (Beyotime, China) according to the manufacturer's instructions. Briefly, fresh mycelia were collected from the rice straw plate covered with cellophane at 72 h, weighed, homogenized, and then centrifuged at 12,000 rpm for 5 min at 4 °C. The supernatants were mixed with ATP detection working dilution in a white 96-well plate. Luminance (RLU) was measured using a SpectraMax® i3x microplate reader (Molecular Devices, Sunnyvale, CA, USA). Standard curves were also generated, and the ATP content of each treatment was calculated. Total ATP levels were expressed as nmol·g^−1^ mycelia. As for the determination of MMP, the fresh mycelium tissues were measured using the JC-10 Assay Kit (Solarbio, Shanghai, China). Fresh mycelial tissues were incubated with 0.5 mL JC-10 working solution at 37 °C for 20 min. Subsequently, the samples were washed at least three times with a washing buffer. Cleaned mycelial tissues were directly captured using a confocal fluorescence microscope (TCS SP8, Leica, Germany) coupled with a digital camera. Simultaneously, the fluorescence intensity of stained mycelial tissues was measured using the SpectraMax® i3x microplate reader (Molecular Devices, Sunnyvale, California, USA).

### Construction of the ATP sensor and lignocellulases-GFP fractions

FRET-based ATP sensor contained an ε subunit to connect the variants of CFP (mseCFP) and YFP (cp173-mVenus) and could monitor the ATP levels in the cytosol or mitochondria independently [38]. Here, a new FRET-based ATP sensor based on the ε subunit from the bacterial F0F1-ATP synthase was constructed to track the real-time content of ATP in different mutants (wt-ATP, Δ*Tgparp*-ATP). The pDR-GW AT1.03YEMK plasmid (http://n2t.net/addgene:28003; RRID: Addgene_28003) contains the functional fragment of the ATP sensor, which was obtained via PCR. Subsequently, additional functional fragments were PCR amplified from the *T. guizhouense* genome, including the upstream region of ura3, the ORF fragment of *ura3*, the downstream region of *ura3*, and a strong promoter fragment. These fragments were then fused together using fusion PCR to generate a long fragment designated as *ura3*-*HygB*-*ura3* upstream-promoter-ATP sensor-*ura3* downstream, and it was transformed into the protoplasts of different mutants through the polyethylene glycol (PEG)-mediated protoplast transformation procedure [63]. Due to the presence of *ura3* in the upstream region of the inserted fragment, there were repetitive sequences between the inserted fragment and the genome, inducing DNA repair and resulting in the loss of the segment between the repetitive sequences. Exploiting this feature, we were able to simultaneously insert the ATP sensor gene into the genome while knocking out the *ura3* gene. Consequently, the *HygB* gene was lost along with it, yielding a *ura3*-deficient FRET sensor strain for subsequent genetic editing. The *ura3* gene encodes orotidine-5'-phosphate decarboxylase, converting 5-fluoroorotic acid to a toxic form (5-fluorouracil). Thus, the *ura3*-deficient transformation was selected using 5-fluoroorotic acid [64].

For the generation of lignocellulases-GFP fusions proteins, the DNA fractions of the representative lignocellulase genes, including EGL (endo-1,4-β-glucanase, OPB37031), CBH (cellobiohydrolase, OPB45635), BGL (β-glucosidase, KKP01743), and XYL (endo-1,4-β-xylanase, OPB45659) were amplified with *T. guizhouense* DNA as template, and the GFP fragment was amplified using plasmid pEGFP-N1 as template (Clontech, USA). The DNA fractions of different lignocellulase genes were ligated with the GFP fragment using the overlapping-PCR technique based on the instructions of CloneAmp HiFi PCR Premix (Clontech, USA). Mutants of *egl-gfp*, *cbh-gfp*, *bgl-gfp*, and *xyl-gfp* were obtained through homologous recombination based on wt and Δ*Tgparp*.

### Confocal imaging and fluorescence intensity analysis of the protoplasts

The protoplast extraction of the lignocellulases-GFP fusion strains was performed according to the method reported by Li et al. [65]. Briefly, GFP fusion strains were cultured in the rice straw medium at 37 °C for 19 h to obtain the fresh mycelia, resuspended with 5 mL of lysing enzymes solution. A 100 mL lysing enzymes solution system contains 750 mg lysing enzyme (L1412, Sigma-Aldrich, USA), 21.86 g sorbitol, and 1.36 g KH_2_PO_4_, and the pH was adjusted to 5.50. The mixture was incubated at 37 °C at 100 rpm for 2 h. The release of protoplasts during the incubation was monitored on a confocal fluorescence microscope (TCS SP8, Leica, Germany) with a 60 × water immersion objective, and GFP was excited at 488 nm and emitted at 510 nm. The fluorescence of the protoplast samples was detected on flow cytometry (BD FACSAria SORP, Becton–Dickinson, USA). Based on the forward scatter characteristics of the protoplasts, an initial scatter gate was applied to collect data from individual cells. GFP protein was excited using a 488 nm laser, and fluorescence signals were detected using a 528/29 bandpass filter. Flow cytometry analyzed 20,000 cells per sample, and gates were set based on differences in fluorescence values to define the positive control expressing GFP [66]. The data were collected and analyzed using the FlowJo software (Version 7.6.1, FlowJo LLC, Ashland, Oregon).

### Confocal imaging and fluorescence intensity analysis of ATP sensor and lignocellulases-GFP

Four-day-old mycelia were collected from the rice straw medium at 37 °C. For ER staining, fresh hyphae were incubated with ER-Tracker™ Red (ThermoFisher, Cat.M7512, Ex/Em = 587 nm/615 nm) at a concentration of 100 nM at room temperature for 5 min in darkness, and immediately washed three times with PBS buffer (pH 7.2). Confocal imaging of the ATP sensor was recorded on a confocal fluorescence microscope (TCS SP8, Leica, Germany) using a 60 × oil immersion objective. Excitation was provided at 435 nm, and emissions were at 475 nm and 527 nm, respectively. Data were presented as the ratio of signal intensities with a ratio of 527 nm to 475 nm. For confocal imaging of lignocellulases-GFP, mycelia were detected on a confocal fluorescence microscope (TCS SP8, Leica, Germany) with a 60 × water immersion objective, and GFP was excited at 488 nm and emitted at 510 nm.

All fluorescences were measured in ImageJ software. Briefly, image colors were extracted through Image-Color-Split Channels, and the RGB format was divided into 8-bit. Auto-threshold was applied to adjust the threshold to avoid exceeding limits, and the intensity analysis was performed by plot profile as previously described by Zhao et al. [41]. Data were completed as at least three independent biological replicates. Statistical data are expressed as means ± standard errors (SE) from all valid pixels.

### Western blot

Different lignocellulases-GFP fusion strains were inoculated into the rice straw medium. After 72 h, the fungal mycelia were collected, and ER-enriched fractions were separated and isolated by Endoplasmic Reticulum Isolation Kit according to the manufacturer's instructions (ER0100, Sigma). SDS-PAGE sample buffer was employed to elute proteins, and together with the supernatant from the fourth day of SSF was used for GFP immunoblot analysis. For GFP-tagged protein detection, the concentrations of ER proteins and secreted proteins were adjusted to the same level (1 mg·mL^−1^) by the BCA protein assay kit (P0009, Beyotime, China), and the samples were separated by 12% SDS-PAGE and transferred to the PVDF membrane (Millipore). GFP monoclonal antibody (GF28R) (MA5-15256, Thermo Fisher) and Goat anti-Mouse IgG (H + L) Highly Cross-Adsorbed Secondary Antibody Alexa Fluor Plus 488 (A32723, Thermo Fisher) were used for immunoblot detection. GFP monoclonal antibody served as the primary antibody (1:2000), and anti-mouse IgG served as the secondary antibody (1:20,000). The Odyssey Infrared Imaging System (version 2.1) was used to detect and analyze the immunoblot signals.

### Supplementary Information

**Additional file 1: Fig. S1**. Growth and straw utilization efficiency of *T. guizhouense* by using rice straw as sole carbon sources at different temperatures. (a) Growth of *T. guizhouense* on plates at different temperatures using rice straw as sole carbon sources. (b) Scanning electron micrographs of the substrates including CK (raw material), T28 and T37, respectively. **Fig. S2.** Quality control of SWATH analysis results. (a) Experimental design to compare the extracellular proteins extracted from *T. guizhouense* at different temperatures. (b) The distribution of proteins' unique peptides number achieved the data analysis quality. (c) The length of identified peptides. (d) Classification of the SWATH-quantified proteins according to the biological function. (e) Distribution of proteins ratio of each sample. (f) Correlation of the quantitative values for all the identified proteins between replicated injections. **Fig. S3.** Model of the critical proteins for the lignocellulose utilization based on the SWATH and PPI analysis. PPI analysis predicted five proteins were considered critical during the lignocellulose utilization process. (a) The PPI network was constructed based on the STRING database between different treatments. (b) Proposed model of *T. guizhouense* in the lignocellulose utilization at different temperature. By compared with T28, four downregulated proteins including Nonribosomal peptide synthetases (NRPS protein, OPB39084), delta-1-pyrroline-5-carboxylate dehydrogenase (P5CDH, OPB43669), aldehyde dehydrogenase (NAD^+^) (ALDH, KKP01310), and ADPRase (OPB41268) and one up-regulated protein (PARP, OPB37503) were deemed as pivotal in T37. NRPS was the modular protein that produced peptide antibiotics and siderophores. P5CDH preferentially used NAD^+^ as a coenzyme to transform glutamate semialdehyde into glutamate. ALDH, a nicotinamide adenine dinucleotide (phosphate) (NAD(P))-dependent enzyme, was involved in detoxification, biosynthesis, antioxidant functions, and structural and regulatory mechanisms. ADPRase and PARP were also involved in the metastasis of ADPR. Intracellular free ADP‐ribose was a highly reactive and potentially toxic molecule. Activation of ADPRase or inhibition of PARP could relieve ER stress by increasing cellular ATP levels. AMP and ribose 5‐phosphate (Ribose 5‐P) might be beneficial for nucleotide recycling, resulting in the suppression of the over-consumption of NAD^+^ and ATP. **Fig. S4.** Knockout or over-expression of different functional genes in *T. guizhouense*. (a) Schematic diagram for genes disruption through double crossover recombination. (b) Schematic diagram for gene over-expression. (c) Diagram of construction principle for preparing the mutants of lignocellulases-GFP fusion strains. HA1 and HA2 were used as two arms of homologous recombination and the *HygB* gene was used as the biomarker for screening. (d) Analysis of expression level of *Tgadprase* gene relative to *Tef* in wt and OE-*Tgadprase* strain determined by qPCR. Data were calculated from three biological replicates. Error bars represent ± SDs. *** *P* < 0.001. A *P*-value < 0.05 is regarded as statistically significant. The expression value was normalized to wt. (e–f) Verification of Δ*Tgparp* (left) and Δ*Tgire* (right) by PCR to verify homologous recombination and whether gene exists. (g) Growth of mutants (Δ*Tgire* and Δ*Tgire*Δ*Tgparp*) inoculated on PDA at 28 °C (top row) and rice straw medium at 37 °C (bottom row), respectively. (h) Verification of Δ*Tgire*Δ*Tgparp* by PCR to verify homologous recombination and whether gene exists. (i) Verification of recombinant strains with GFP labeling by PCR using two relevant primer pairs of E-*egl* (E-*cbh*, E-*blg*, and E-*xyl*) and E-GFP to verify homologous recombination. **Fig. S5.** Southern blot analysis of gene *Tgparp*. The genomic DNA was digested with the restriction enzymes and hybridized with the probes amplified by primers listed in Table. S2. Lane M: DNA marker; Lane 1: wt-1; Lane 2: Δ*Tgparp*-1; Lane 3: positive plasmid-1; Lane 4: wt-2; Lane 5: Δ*Tgparp*-2; Lane 6: Positive plasmid-2. **Fig. S6.** The profile of carbon utilization using Biolog FF Microplates at 28 °C. Three independent biological experiments were performed and the data were collected. Error bars represent ± SDs. **Fig. S7.** Transcriptional level of representative lignocellulase genes in wt and Δ*Tgparp* at 37 °C. Data were calculated from three biological replicates. Error bars represent ± SDs. * *P* < 0.05, ** *P* < 0.01. A *P-*value < 0.05 was regarded as statistically significant. The expression values are normalized to wt. Endoglucanase genes: 1–5 (A1A106567.1, A1A101831.1, A1A107632.1, A1A104288.1, and A1A110611.1); cellobiohydrolase genes: 6–9 (A1A104298.1, A1A108865.1, A1A104556.1, and A1A102650.1); glucosidase genes: 10–13 (A1A110026.1, A1A105994.1, A1A107778.1, and A1A111035.1); hemicellulase and auxiliary hydrolyzing genes: 14–19 (A1A111258.1, A1A111547.1, A1A102817.1, A1A112191.1, A1A105029.1, and A1A100991.1). **Fig. S8.** The ATP level between wt and Δ*Tgparp* with the gradual increase of tempeature (from 28 °C to 37 °C). Three independent biological experiments were performed and data was collected. Error bars represent ± SDs. ** *P* < 0.01, ** *P* < 0.001. A *P-*value < 0.05 was regarded as statistically significant. **Fig. S9.** Growth of *T. guizhouense* inoculated at 37 °C for 48 h when different concentrations of exogenous ATP-Na_2_ were added. **Fig. S10.** Construction and growth of strains with the ATP sensor. (a) Schematic of the ATP sensor. (b) Equally harvested biomass of strains wt-ATP and Δ*Tgparp*-ATP were inoculated on PDA at 28 °C (left) and rice straw medium at 37 °C (right), respectively. (c) Verification of the strains wt-ATP and Δ*Tgparp*-ATP through PCR by using two relevant primer pairs of E-ATP-F and E-ATP-R to verify the ATP sensor fragment, and E3 was used to verify the existence of gene *Tgparp*. **Fig. S11.** Flow cytometry dot plot of the protoplasts containing the lignocellulases-GFP at different treatments under heat stress. The lignocellulases-GFP fusion strains contained *egl-gfp* (a), *cbh-gfp* (b), *bgl-gfp* (c), and *xyl-gfp* (d); the dot plot of wt is represented in blue, while Δ*Tgparp* are depicted in red. **Fig. S12.** Growth condition and lignocellulose utilization efficiency of RNAi-*Tgparp* under heat stress by knocking down of *Tgparp* through the RNA interference technology. (a) Schematic diagram for the generation of RNAi-*Tgparp*. MCS1: Sense strand of gene *Tgparp*, MCS2: Antisense strand of gene *Tgparp*. (b) Relative expression of gene *Tgparp* between wt, RNAi-*Tgparp*-1, and RNAi-*Tgparp*-2. The expression value is normalized to wt. (c) Biomass of wt, RNAi-*Tgparp*-1 and RNAi-*Tgparp*-2 inoculated on PDA (top row) and rice straw medium (bottom row), respectively. (d) Comparison of the lignocellulolytic activities including FPA, EG, CBH, and XYL between wt, RNAi-*Tgparp*-1, and RNAi-*Tgparp*-2 under heat stress. **(e)** Relative expression of genes related to DNA repair between wt, Δ*Tgparp* and RNAi-*Tgparp*-1. Data were calculated from three biological replicates. Error bars represent ± SDs. ** *P* < 0.01, *** *P* < 0.001. A *P*-value < 0.05 is regarded as statistically significant. **Fig. S13.** Construction and growth of strains with NAD^+^ sensor. (a) Schematic of the ATP sensor. (b) The growth condition of wt with mCherry-cpYFP / mCherry-FiNad and Δ*Tgparp* with mCherry-cpYFP / mCherry-FiNad inoculated on PDA at 28 °C (top row) and rice straw medium at 37 °C (bottom row), respectively. **(c)** Verification of the strains that successfully expressed the mCherry-cpYFP or mCherry-FiNad in wt and Δ*Tgparp*. **Fig. S14.** Confocal imaging and fluorescence intensity analysis of NAD^+^ in *T. guizhouense*. (a-b) Confocal imaging analysis of hyphae from wt-mCherry-cpYFP (left) and Δ*Tgparp*-mCherry-cpYFP (right) under heat stress, bar = 3 μm. (c-d) Confocal imaging analysis of hyphae from wt-mCherry-FiNad (left) and Δ*Tgparp*-mCherry-FiNad (right) under heat stress, bar = 3 μm. (e–h) Fluorescence intensity of the two channels (mCherry and cpYFP) detected in different strains. (i-l) Green/Red ratios plotted for single pixels of the mycelium with the mCherry-cpYFP or mCherry-FiNad in wt and Δ*Tgparp*. Blue numbers represent the average of all values. (m–n) Green/Red ratio of mCherry-cpYFP or mCherry-FiNad in wt and Δ*Tgparp* from more mycelia repeats. The box plot means the median values of ratios. (o) Ratio of mCherry-FiNad/mCherry-cpYFP between wt and Δ*Tgparp* according to the results in graphs m and n. (p) Biochemical analysis of cellular NAD^+^ content under heat stress between wt and Δ*Tgparp*. **Table S1.** SWATH quantifies proteins of *Trichoderma guizhouense* NJAU4742 at different temperatures using straw as the only carbon source. **Table S2.** PCR primers used in this study.

## Data Availability

All data supporting the findings of this study are available within the paper and its additional materials. Source data that support the findings of this study are available from the corresponding author on reasonable request.
