# Supplementary material for: Cellular ATP redistribution achieved by deleting Tgparp improves lignocellulose utilization of Trichoderma under heat stress
Source: Biotechnol Biofuels Bioprod. 2024 Apr 18;17:54. doi: 10.1186/s13068-024-02502-8 (PMC11027231; doi:10.1186/s13068-024-02502-8)
Supplement: Supplementary file 1 — Additional file 1: Fig. S1. Growth and straw utilization efficiency of T. guizhouense by using rice straw as sole carbon sources at different temperatures. (a) Growth of T. guizhouense on plates at different temperatures using rice straw as sole carbon sources. (b) Scanning electron micrographs of the substrates including CK (raw material), T28 and T37, respectively. Fig. S2. Quality control of SWATH analysis results. (a) Experimental design to compare the extracellular proteins extracted from T. guizhouense at different temperatures. (b) The distribution of proteins' unique peptides number achieved the data analysis quality. (c) The length of identified peptides. (d) Classification of the SWATH-quantified proteins according to the biological function. (e) Distribution of proteins ratio of each sample. (f) Correlation of the quantitative values for all the identified proteins between replicated injections. Fig. S3. Model of the critical proteins for the lignocellulose utilization based on the SWATH and PPI analysis. PPI analysis predicted five proteins were considered critical during the lignocellulose utilization process. (a) The PPI network was constructed based on the STRING database between different treatments. (b) Proposed model of T. guizhouense in the lignocellulose utilization at different temperature. By compared with T28, four downregulated proteins including Nonribosomal peptide synthetases (NRPS protein, OPB39084), delta-1-pyrroline-5-carboxylate dehydrogenase (P5CDH, OPB43669), aldehyde dehydrogenase (NAD+) (ALDH, KKP01310), and ADPRase (OPB41268) and one up-regulated protein (PARP, OPB37503) were deemed as pivotal in T37. NRPS was the modular protein that produced peptide antibiotics and siderophores. P5CDH preferentially used NAD+ as a coenzyme to transform glutamate semialdehyde into glutamate. ALDH, a nicotinamide adenine dinucleotide (phosphate) (NAD(P))-dependent enzyme, was involved in detoxification, biosynthesis, antioxidant functions, and str [file 13068_2024_2502_MOESM1_ESM.docx]

**Supplementary Materials for**

**Cellular ATP redistribution achieved by deleting *Tgparp* improves lignocellulose utilization of *Trichoderma* under heat stress**

Tuo Li^1, 2^, Yang Liu^1, 2^, Han Zhu^1, 2^, Linhua Cao^1, 2^, Yihao Zhou^1, 2^, Dongyang Liu^1, 2^[[1]](#footnote-1)^*^, Qirong Shen^1, 2^

**Affiliations**

1. Key lab of organic-based fertilizers of China and Jiangsu provincial key lab for solid organic waste utilization;

2. Nanjing Agricultural University, Nanjing 210095, Jiangsu, China

***Corresponding authors:**

**Dongyang Liu**, College of Resources & Environmental Sciences, Nanjing Agricultural University, 210095, Nanjing, P. R. China E-mail: [liudongyang@njau.edu.cn](mailto:liudongyang@njau.edu.cn).

**This PDF file includes:**

Supplementary Figure S1-S14

Supplementary Table S1-S2

Supplementary Methods 11

References 10

# 1. Supplementary Figures

**
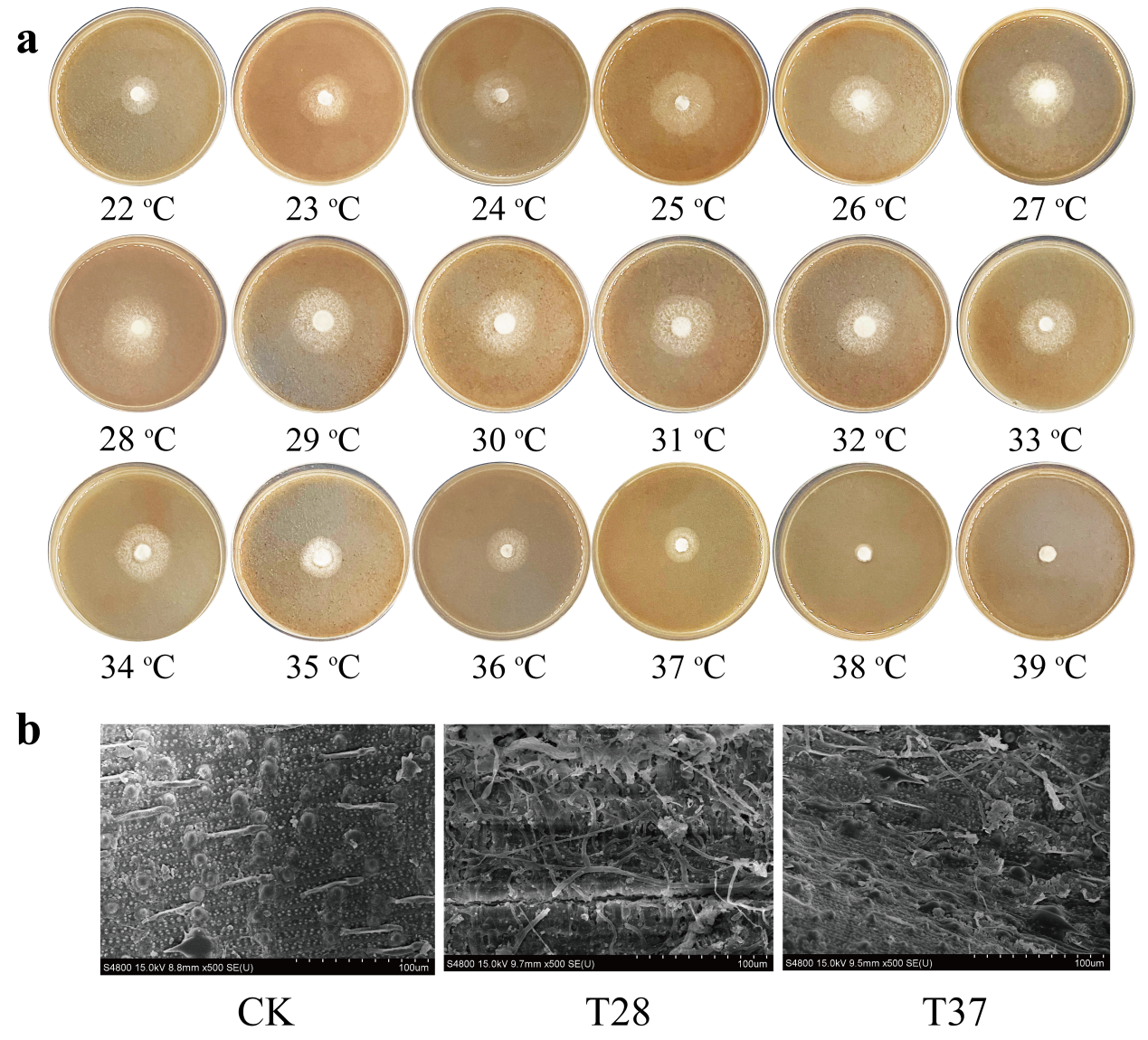
**

**Fig. S1 Growth and straw utilization efficiency of *T. guizhouense* by using rice straw as sole carbon sources at different temperatures. (a)** Growth of *T. guizhouense* on plates at different temperatures using rice straw as sole carbon sources. **(b)** Scanning electron micrographs of the substrates including CK (raw material), T28 and T37, respectively.

**
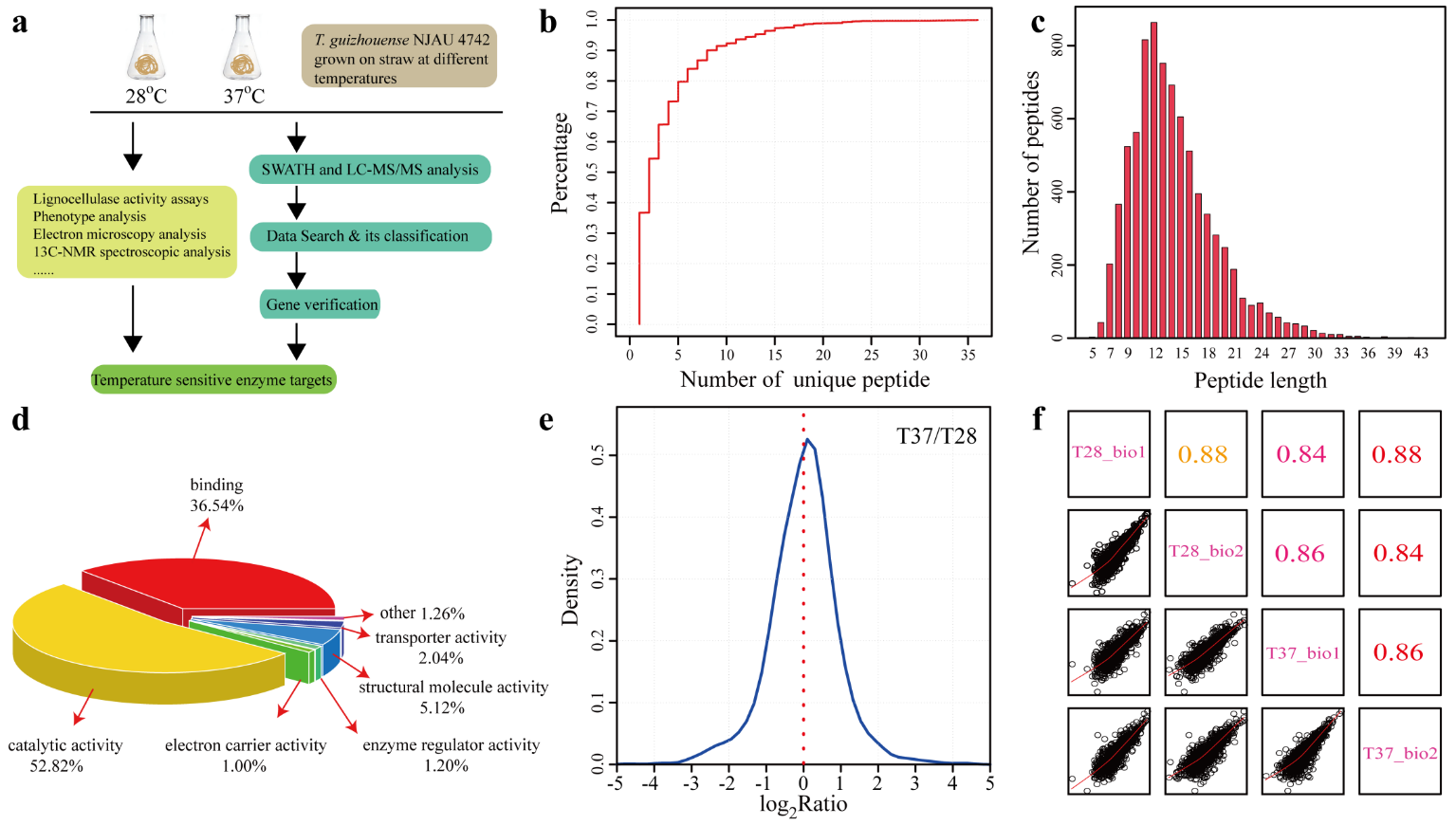
**

**Fig. S2 Quality control of SWATH analysis results. (a)** Experimental design to compare the extracellular proteins extracted from *T. guizhouense* at different temperatures. **(b)** The distribution of proteins' unique peptides number achieved the data analysis quality. **(c)** The length of identified peptides. **(d)** Classification of the SWATH-quantified proteins according to the biological function. **(e)** Distribution of proteins ratio of each sample. **(f)** Correlation of the quantitative values for all the identified proteins between replicated injections.


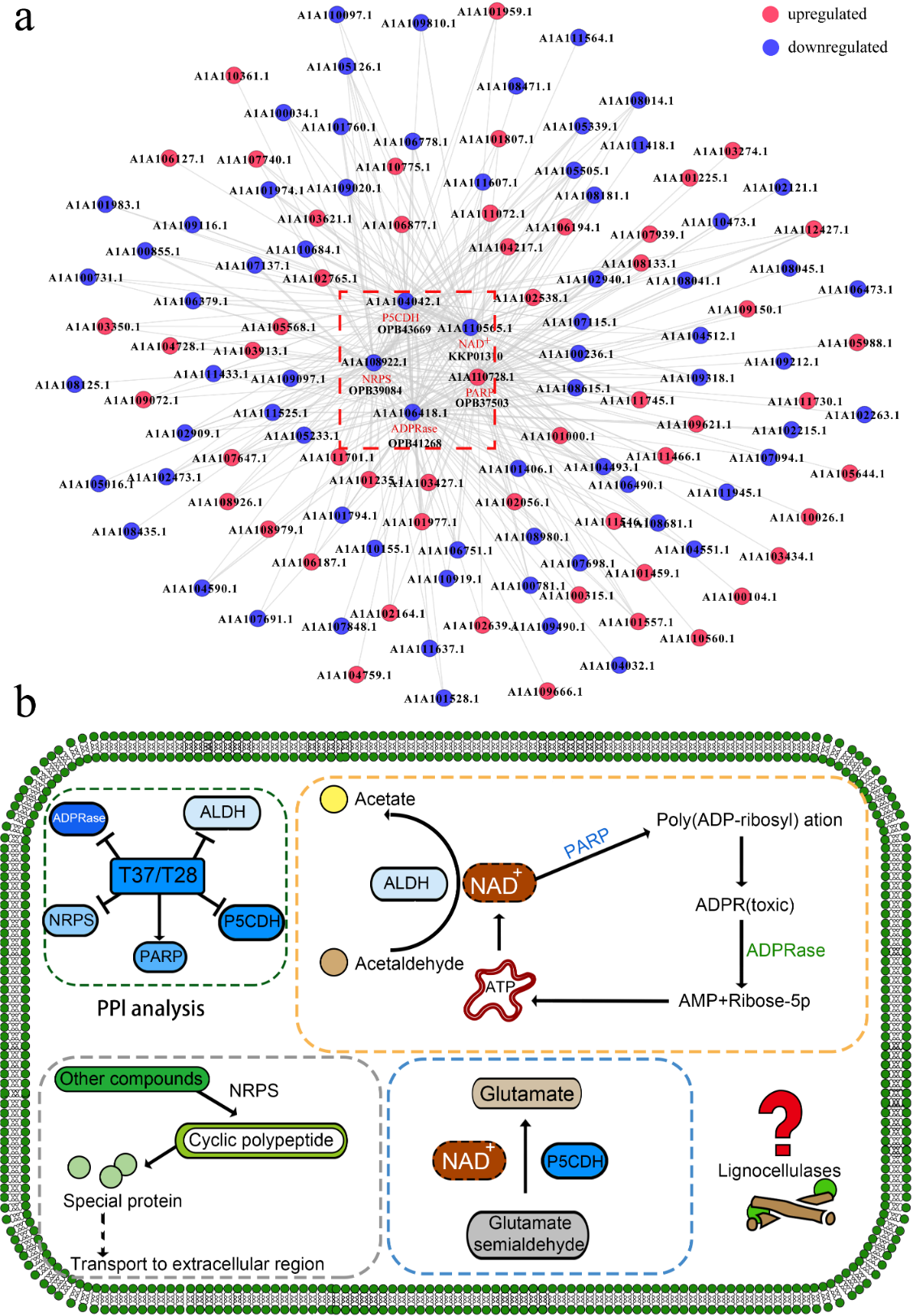


**Fig. S3** **Model of the critical proteins for the lignocellulose utilization based on the SWATH and PPI analysis.** PPI analysis predicted five proteins were considered critical during the lignocellulose utilization process. **(a)** The PPI network was constructed based on the STRING database between different treatments. **(b)** Proposed model of *T. guizhouense* in the lignocellulose utilization at different temperature. By compared with T28, four down-regulated proteins including Nonribosomal peptide synthetases (NRPS protein, [OPB39084](https://www.ncbi.nlm.nih.gov/protein/OPB39084.1?report=genbank&log$=protalign&blast_rank=1&RID=W2U4EPKC01R)), delta-1-pyrroline-5-carboxylate [dehydrogenase](https://www.sciencedirect.com/topics/biochemistry-genetics-and-molecular-biology/dehydrogenase) (P5CDH, [OPB43669](https://www.ncbi.nlm.nih.gov/protein/OPB43669.1?report=genbank&log$=protalign&blast_rank=1&RID=W2HRY3MJ016)), aldehyde dehydrogenase (NAD^+^) (ALDH, [KKP01310](https://www.ncbi.nlm.nih.gov/protein/KKP01310.1?report=genbank&log$=protalign&blast_rank=1&RID=W2TT3TKZ013)), and ADPRase (OPB41268) and one up-regulated protein (PARP, OPB37503) were deemed as pivotal in T37. NRPS was the modular protein that produced peptide antibiotics and siderophores. P5CDH preferentially used NAD^+^ as a coenzyme to transform glutamate semialdehyde into glutamate. ALDH, a nicotinamide adenine dinucleotide (phosphate) (NAD(P)) dependent enzyme, was involved in detoxification, biosynthesis, antioxidant functions, and structural and regulatory mechanisms. ADPRase and PARP were also involved in the metastasis of ADPR. Intracellular free ADP‐ribose was a highly reactive and potentially toxic molecule. Activation of ADPRase or inhibition of PARP could relieve ER stress by increasing cellular ATP levels. AMP and ribose 5‐phosphate (Ribose 5‐P) might be beneficial for nucleotide recycling, resulting in the suppression of the over-consumption of NAD^+^ and ATP.

**
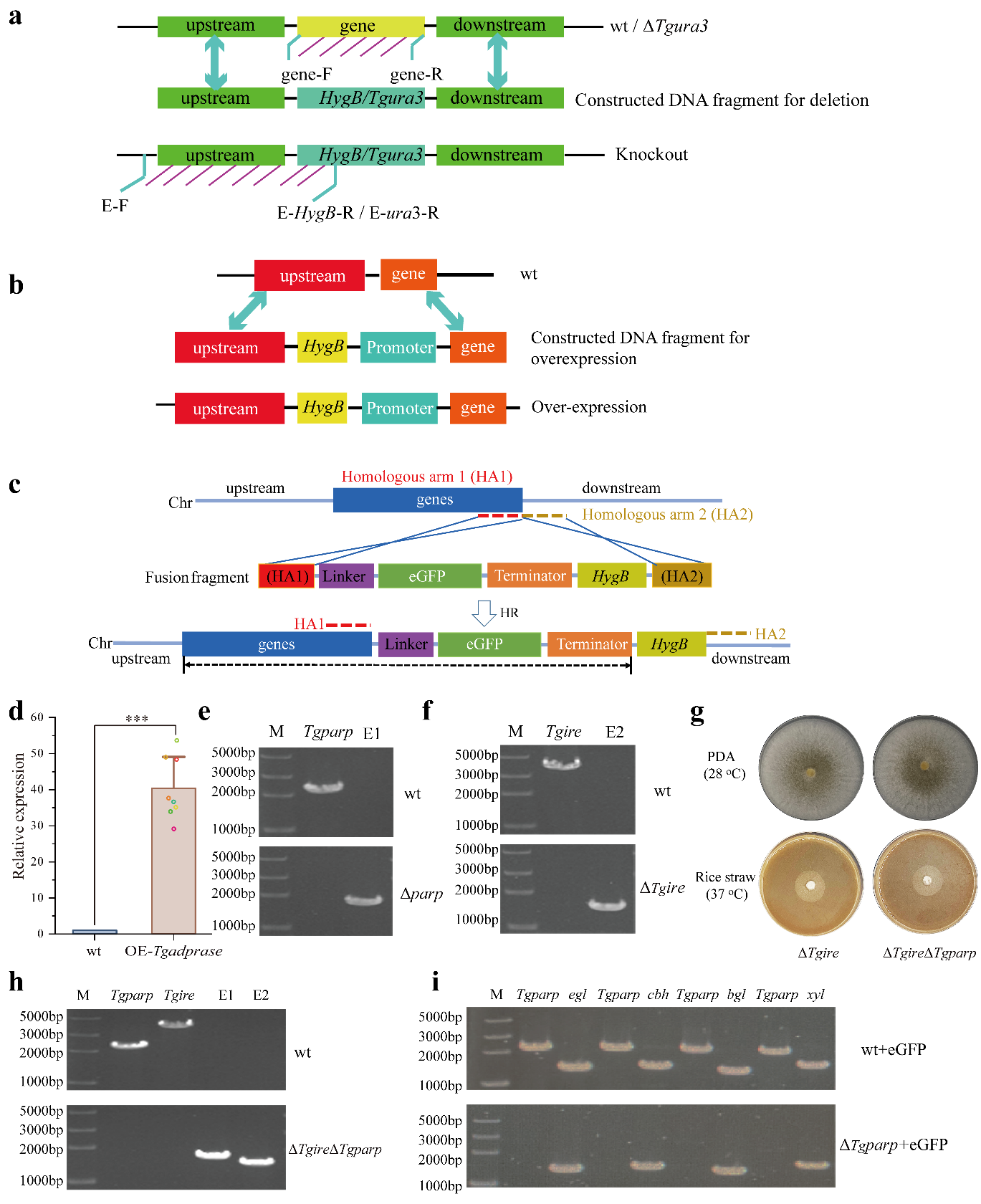
**

**Fig. S4 Knockout or over-expression of different functional genes in *T. guizhouense*. (a)** Schematic diagram for genes disruption through double crossover recombination. **(b)** Schematic diagram for gene over-expression. **(c)** Diagram of construction principle for preparing the mutants of lignocellulases-GFP fusion strains. HA1 and HA2 were used as two arms of homologous recombination and the *HygB* gene was used as the biomarker for screening. **(d)** Analysis of expression level of *Tgadprase* gene relative to *Tef* in wt and OE-*Tgadprase* strain determined by qPCR. Data were calculated from three biological replicates. Error bars represent±SDs. *** *P* < 0.001. A *P*-value < 0.05 is regarded as statistically significant. The expression value was normalized to wt. **(e-f)** Verification of Δ*Tgparp* (left) and Δ*Tgire* (right) by PCR to verify homologous recombination and whether gene exists. **(g)** Growth of mutants (Δ*Tgire* and Δ*Tgire*Δ*Tgparp*) inoculated on PDA at 28 ^o^C (top row) and rice straw medium at 37 ^o^C (bottom row), respectively. **(h)** Verification of Δ*Tgire*Δ*Tgparp* by PCR to verify homologous recombination and whether gene exists. **(i)** Verification of recombinant strains with GFP labeling by PCR using two relevant primer pairs of E-*egl* (E-*cbh*, E-*blg*, and E-*xyl*) and E-GFP to verify homologous recombination.

**
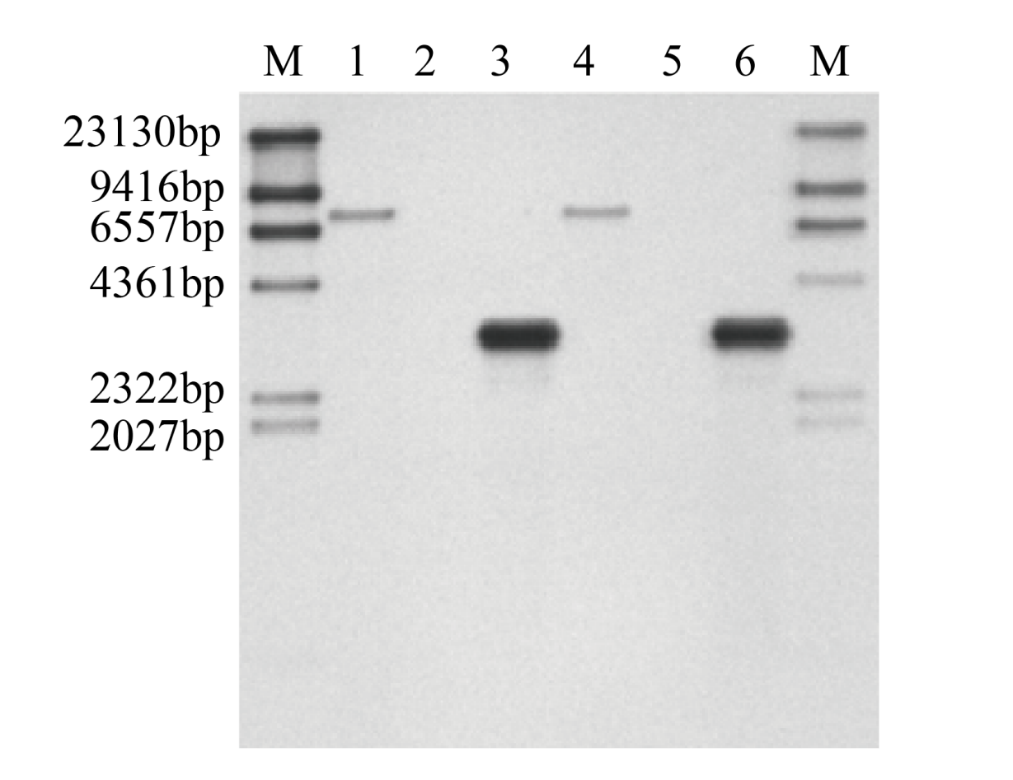
**

**Fig. S5 Southern blot analysis of gene *Tgparp*.** The genomic DNA was digested with the restriction enzymes and hybridized with the probes amplified by primers listed in Table. S2. Lane M: DNA marker; Lane 1: wt-1; Lane 2: Δ*Tgparp*-1; Lane 3: positive plasmid-1; Lane 4: wt-2; Lane 5: Δ*Tgparp*-2; Lane 6: Positive plasmid-2.


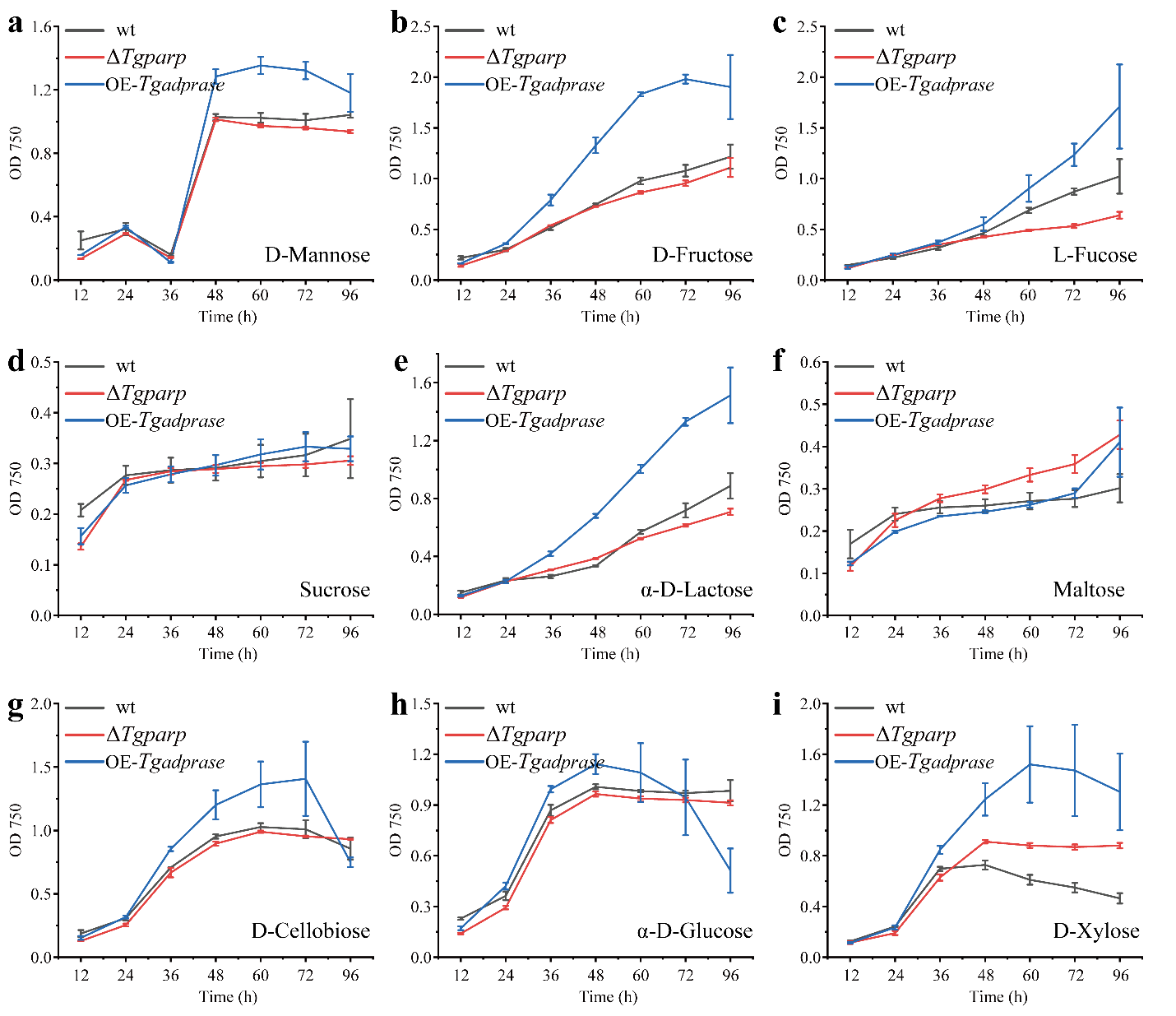


**Fig. S6 The profile of carbon utilization using Biolog FF Microplates at 28 ^o^C.** Three independent biological experiments were performed and the data was collected. Error bars represent±SDs.


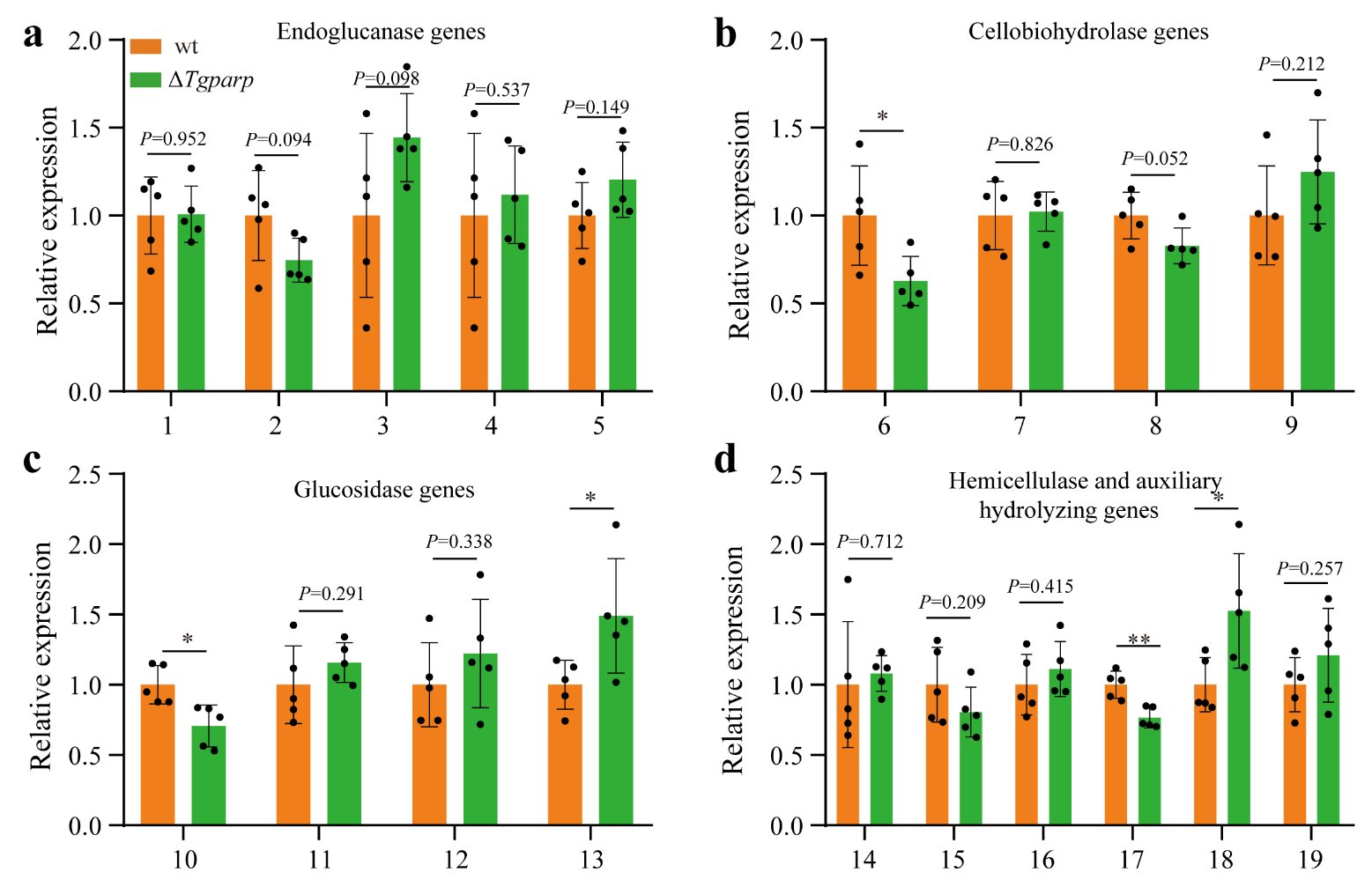


**Fig. S7 Transcriptional level of representative lignocellulase genes in wt and Δ*Tgparp* at 37 ^o^C.** Data were calculated from three biological replicates. Error bars represent ± SDs. * *P* < 0.05, ** *P* < 0.01. A *P-*value < 0.05 was regarded as statistically significant. The expression values are normalized to wt. Endoglucanase genes: 1-5 (A1A106567.1, A1A101831.1, A1A107632.1, A1A104288.1, and A1A110611.1); cellobiohydrolase genes: 6-9 (A1A104298.1, A1A108865.1, A1A104556.1, and A1A102650.1); glucosidase genes: 10-13 (A1A110026.1, A1A105994.1, A1A107778.1, and A1A111035.1); hemicellulase and auxiliary hydrolyzing genes: 14-19 (A1A111258.1, A1A111547.1, A1A102817.1, A1A112191.1, A1A105029.1, and A1A100991.1).

**
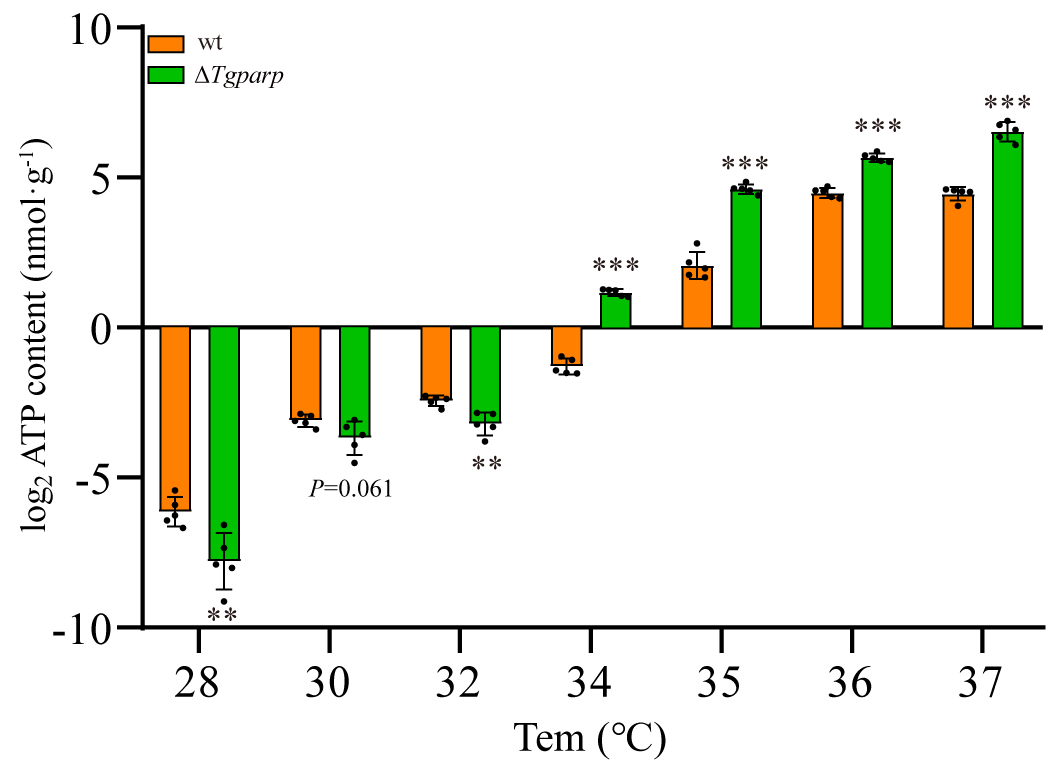
**

**Fig. S8 The ATP level between wt and Δ*Tgparp* with the gradual increase of tempeature (from 28 ^o^C to 37 ^o^C).** Three independent biological experiments were performed and data was collected. Error bars represent ± SDs. ** *P* < 0.01, ** *P* < 0.001. A *P-*value < 0.05 was regarded as statistically significant.

**
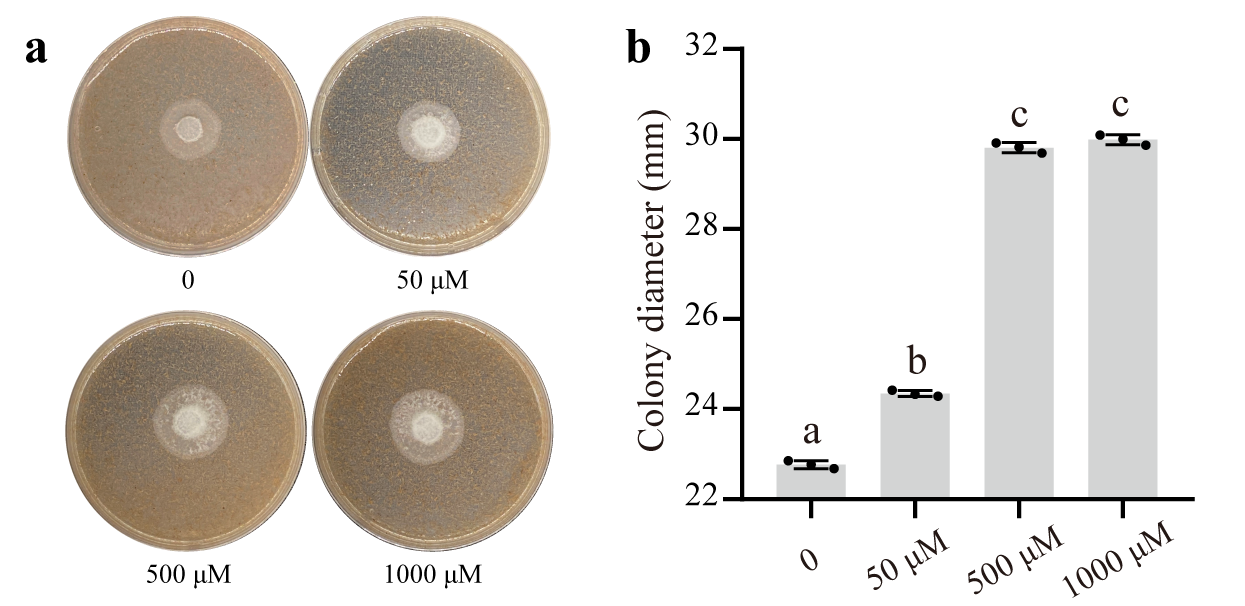
**

**Fig. S9 Growth of *T. guizhouense* inoculated at 37 ^o^C for 48 h when different concentrations of exogenous ATP-Na_2_ were added.**


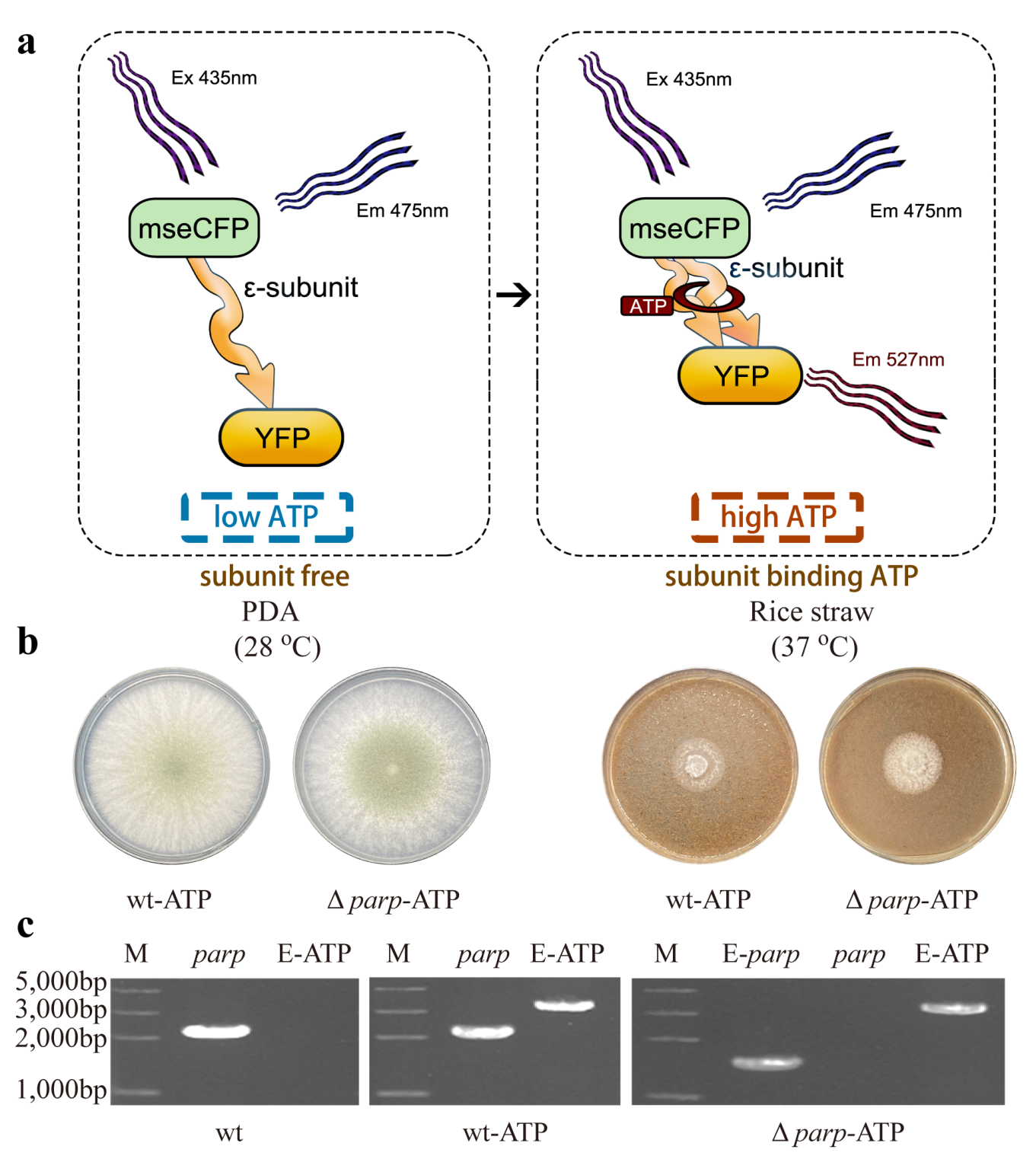


**Fig. S10** **Construction and growth of strains with the ATP sensor.** **(a)** Schematic of the ATP sensor. **(b)** Equally harvested biomass of strains wt-ATP and Δ*Tgparp*-ATP were inoculated on PDA at 28 ^o^C (left) and rice straw medium at 37 ^o^C (right), respectively. **(c)** Verification of the strains wt-ATP and Δ*Tgparp*-ATP through PCR by using two relevant primer pairs of E-ATP-F and E-ATP-R to verify the ATP sensor fragment, and E3 was used to verify the existence of gene *Tgparp*.


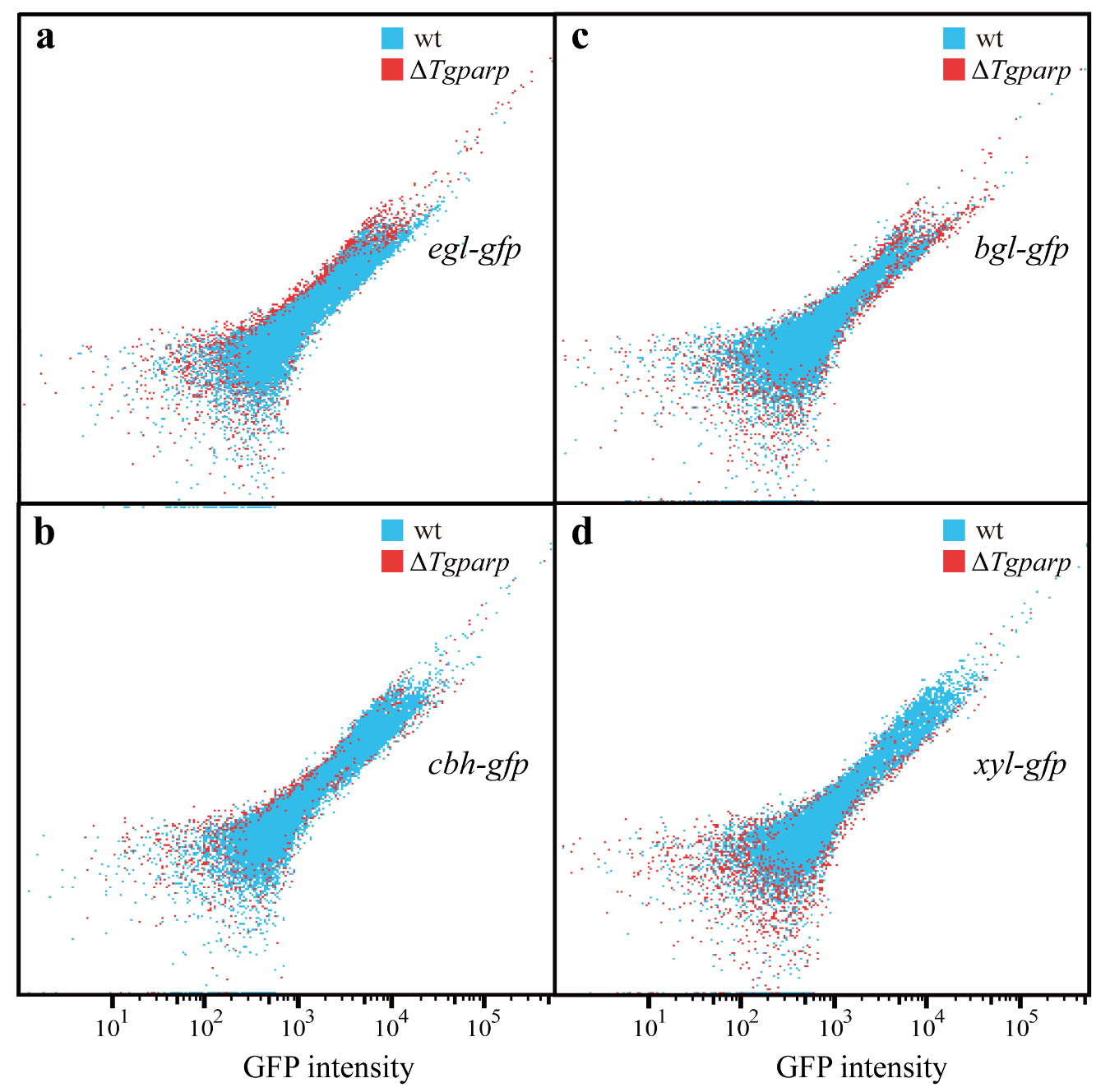


**Fig. S11 Flow cytometry dot plot of the protoplasts containing the lignocellulases-GFP at different treatments under heat stress.** The lignocellulases-GFP fusion strains contained *egl-gfp* (**a**), *cbh-gfp* (**b**), *bgl-gfp* (**c**) and *xyl-gfp* (**d**); the dot plot of wt is represented in blue, while Δ*Tgparp* are depicted in red.


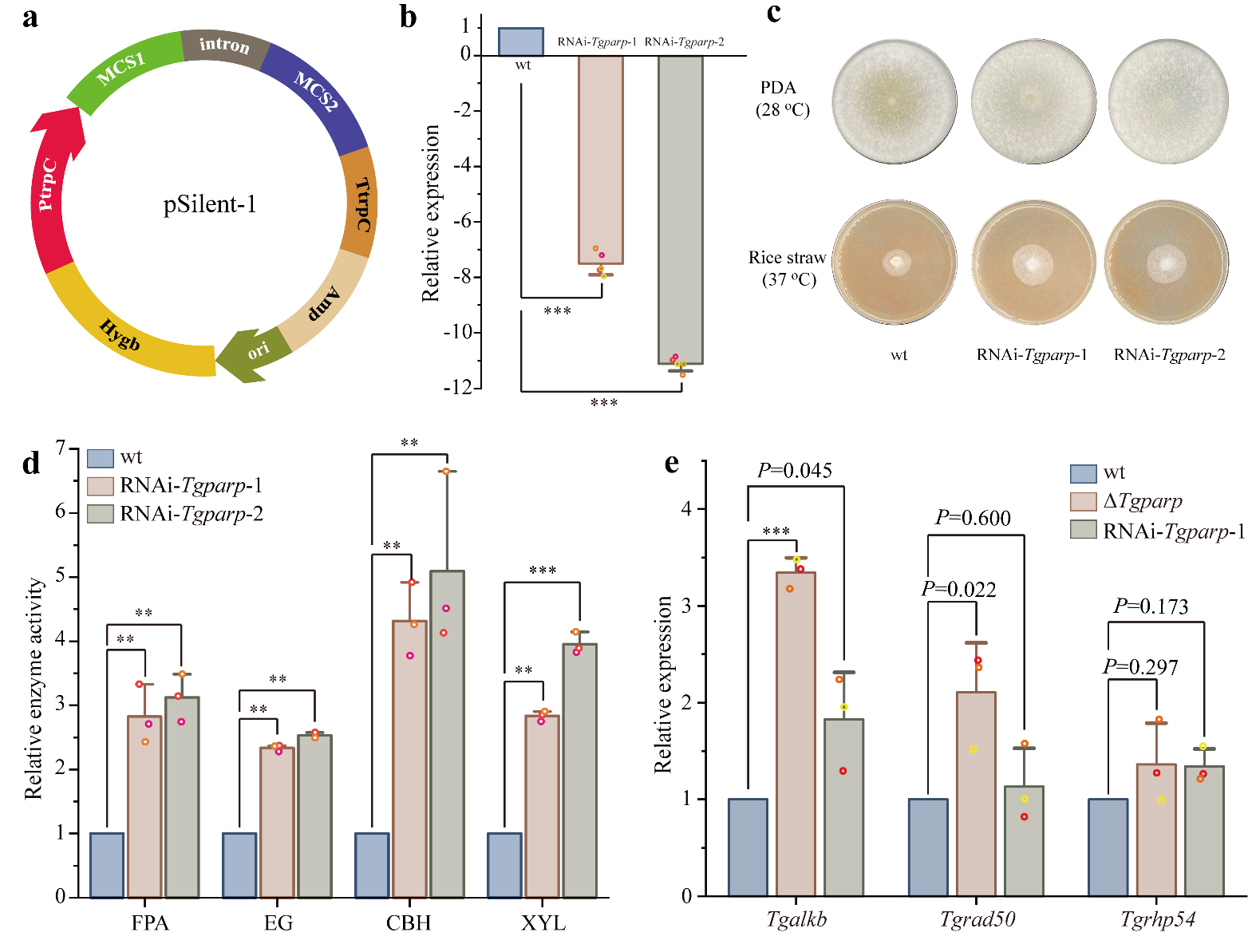


**Fig. S12 Growth condition and lignocellulose utilization efficiency of RNAi-*Tgparp* under heat stress by knocking down of *Tgparp* through the RNA interference technology. (a)** Schematic diagram for the generation of RNAi-*Tgparp*. MCS1: Sense strand of gene *Tgparp*, MCS2: Antisense strand of gene *Tgparp***. (b)** Relative expression of gene *Tgparp* between wt, RNAi-*Tgparp*-1 and RNAi-*Tgparp*-2. The expression value is normalized to wt. **(c)** Biomass of wt, RNAi-*Tgparp*-1 and RNAi-*Tgparp*-2 inoculated on PDA (top row) and rice straw medium (bottom row), respectively. **(d)** Comparison of the lignocellulolytic activities including FPA, EG, CBH and XYL between wt, RNAi-*Tgparp*-1 and RNAi-*Tgparp*-2 under heat stress. **(e)** Relative expression of genes related to DNA repair between wt, Δ*Tgparp* and RNAi-*Tgparp*-1. Data were calculated from three biological replicates. Error bars represent±SDs. ** *P* < 0.01, *** *P* < 0.001. A *P*-value < 0.05 is regarded as statistically significant.


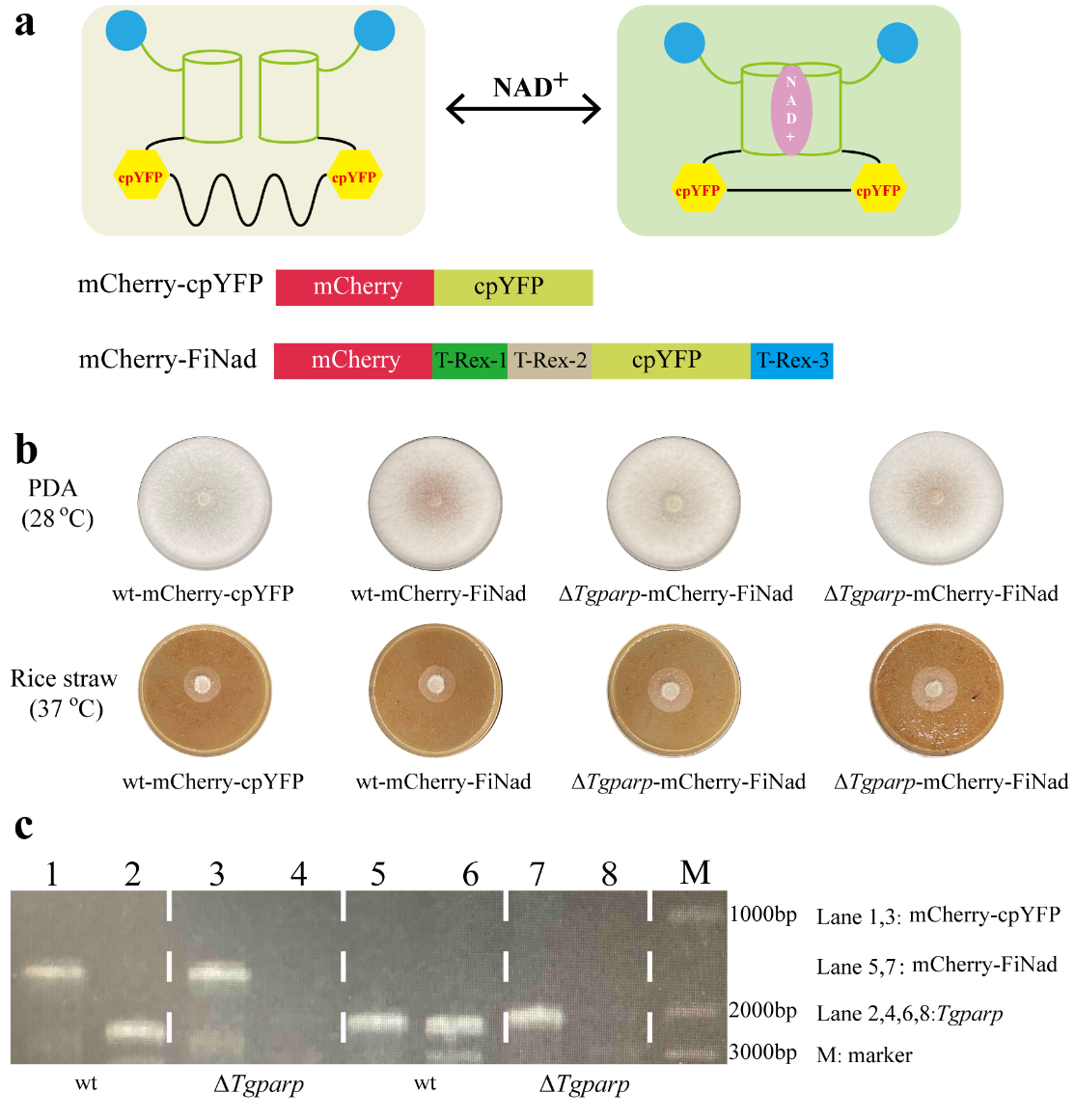


**Fig. S13** **Construction and growth of strains with NAD^+^ sensor. (a)** Schematic of the ATP sensor. **(b)** The growth condition of wt with mCherry-cpYFP / mCherry-FiNad and Δ*Tgparp* with mCherry-cpYFP / mCherry-FiNad inoculated on PDA at 28 ^o^C (top row) and rice straw medium at 37 ^o^C (bottom row), respectively. **(c)** Verification of the strains that successfully expressed the mCherry-cpYFP or mCherry-FiNad in wt and Δ*Tgparp*.


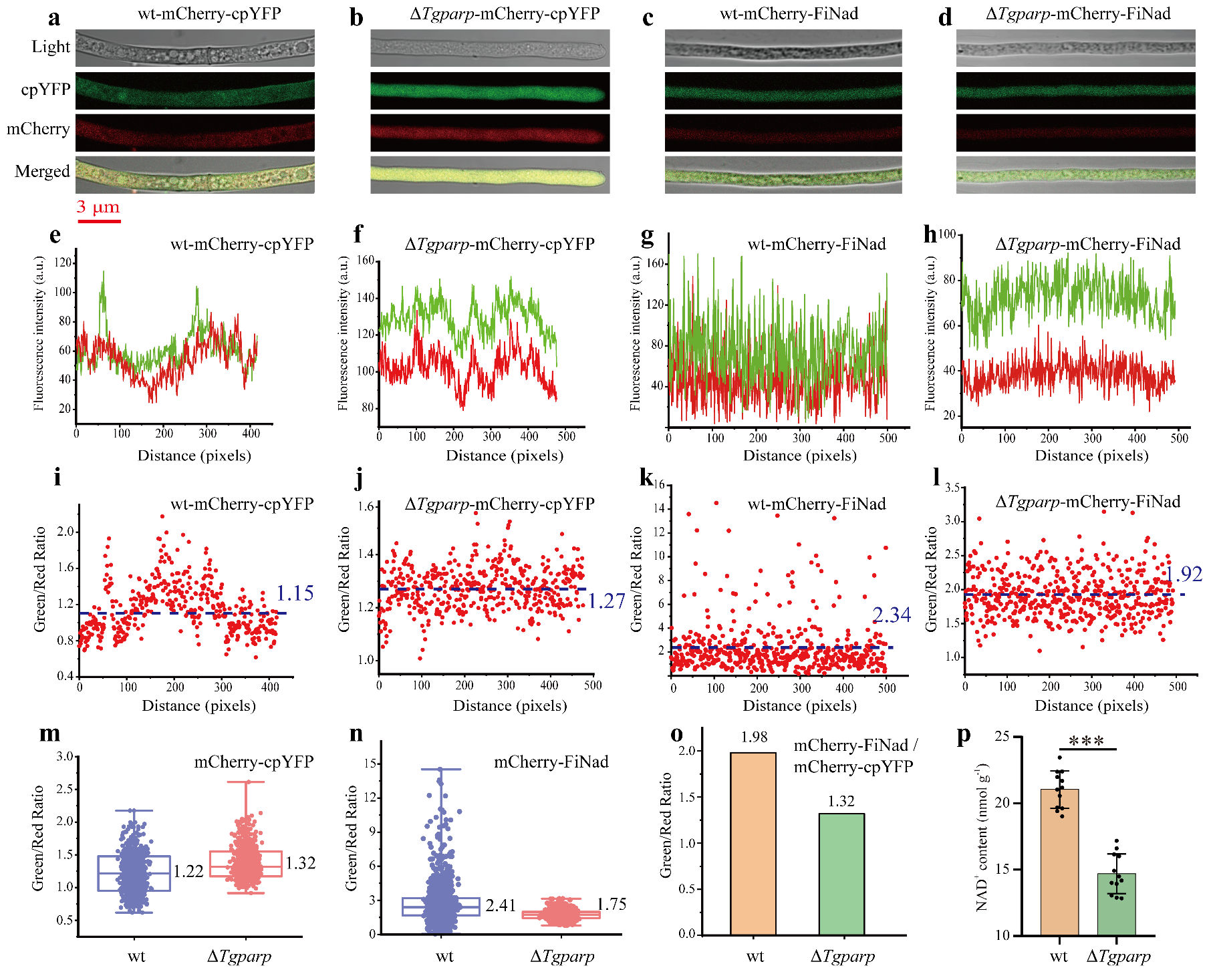


**Fig. S14 Confocal imaging and fluorescence intensity analysis of NAD^+^ in *T. guizhouense*.** **(a-b)** Confocal imaging analysis of hyphae from wt-mCherry-cpYFP (left) and Δ*Tgparp*-mCherry-cpYFP (right) under heat stress, bar=3 μm. **(c-d)** Confocal imaging analysis of hyphae from wt-mCherry-FiNad (left) and Δ*Tgparp*-mCherry-FiNad (right) under heat stress, bar=3 μm. **(e-h)** Fluorescence intensity of the two channels (mCherry and cpYFP) detected in different strains. **(i-l)** Green/Red ratios plotted for single pixels of the mycelium with the mCherry-cpYFP or mCherry-FiNad in wt and Δ*Tgparp*. Blue numbers represent the average of all values. **(m-n)** Green/Red ratio of mCherry-cpYFP or mCherry-FiNad in wt and Δ*Tgparp* from more mycelia repeats. The box plot means the median values of ratios. **(o)** Ratio of mCherry-FiNad/mCherry-cpYFP between wt and Δ*Tgparp* according to the results in graphs m and n. **(p)** Biochemical analysis of cellular NAD^+^ content under heat stress between wt and Δ*Tgparp*.

# 2. Supplementary Tables

**Table S1: SWATH quantifies proteins *Trichoderma guizhouense* NJAU4742 at different temperatures.**

| **Coverage** | **Peptide Number(95%)** | **Accession** | **Name** | **Family** | **T37 / T28** | **SignalP** |
| --- | --- | --- | --- | --- | --- | --- |
| **Splitting of cross-linked glucan chain into single chain**s | | | | | | |
| 0.1012 | 3 | A1A110863.1 | endoglucanase EG-II | GH5 | 1.08 | Y |
| 0.1074 | 3 | A1A104288.1 | endo-1,4-β-glucanase | GH5 | 0.77 | Y |
| 0.1471 | 4 | A1A106567.1 | endo-1,4-β-glucanase | GH12 | 0.12 | Y |
| 0.0455 | 1 | A1A110611.1 | endo-1,3(4)-β-glucanase | GH16 | 1.26 | N |
| 0.0685 | 1 | A1A101062.1 | endo-1,4-β-glucanase | GH5 | 0.52 | Y |
| 0.0258 | 1 | A1A101831.1 | endo-1,4-β-glucanase | GH7 | 0.70 | Y |
| **Hydrolysis of single glucan chain into cellobiose** | | | | | | |
| 0.2614 | 10 | A1A102028.1 | cellobiohydrolase | GH7 | 0.42 | Y |
| 0.2585 | 14 | A1A104298.1 | exoglucanase 2 | GH6 | 0.30 | Y |
| **Conversion of cellobiose into glucose** | | | | | | |
| 0.1665 | 11 | A1A105994.1 | α-glucosidase | GH31 | 0.85 | N |
| 0.3025 | 18 | A1A112192.1 | β-glucosidase BGL1/CEL3a | GH3 | 0.25 | Y |
| 0.4774 | 18 | A1A108471.1 | β-glucosidase BGL2/CEL1a | GH1 | 0.45 | N |
| 0.1688 | 9 | A1A110026.1 | β-glucosidase | GH3 | 0.73 | Y |
| 0.0946 | 6 | A1A111258.1 | β-glucosidase | GH3 | 0.52 | Y |
| **Cellulose-binding and other auxiliary cellulose hydrolyzing proteins** | | | | | | |
| 0.6409 | 21 | A1A102057.1 | endo-1,4-β-xylanase | GH10 | 0.92 | Y |
| 0.2624 | 4 | A1A103578.1 | endo-1,4-β-xylanase | GH11 | 0.16 | Y |
| 0.1643 | 12 | A1A100991.1 | xyloglucanase | GH74 | 0.30 | Y |
| 0.1118 | 4 | A1A102032.1 | endo-β-1,4-xylanase XYN4 | GH30 | 2.13 | Y |
| 0.1037 | 4 | A1A107051.1 | acetylxylan esterase | CBM5 | 0.44 | Y |
| 0.0251 | 1 | A1A102817.1 | xylosidase/arabiNsidase | GH43 | 2.93 | N |
| 0.1937 | 11 | A1A112191.1 | β-xylosidase BXL1 | GH3 | 0.72 | Y |
| 0.1433 | 9 | A1A107682.1 | β-xylosidase XYL3b | GH3 | 0.49 | Y |
| 0.1303 | 5 | A1A111547.1 | xylan 1,4-β-xylosidase | GH43 | 0.42 | N |
| 0.0277 | 2 | A1A106576.1 | trehalase | GH37 | 0.15 | Y |
| 0.0389 | 1 | A1A105029.1 | mannanase | GH5 | 0.42 | Y |
| 0.1331 | 2 | A1A106103.1 | CIP1 protein | CBM1 | 0.38 | Y |
| 0.0917 | 4 | A1A101990.1 | exochitinase | GH20 | 0.77 | Y |
| 0.2302 | 7 | A1A104090.1 | endochitinase | GH18 | 0.36 | Y |
| 0.0453 | 3 | A1A111962.1 | putative chitinase | GH18 | 0.25 | N |
| 0.3422 | 22 | A1A107504.1 | α-glucuronidase | GH67 | 0.84 | N |
| 0.0514 | 6 | A1A100286.1 | glycogen debranching enzyme | GH13 | 0.70 | N |
| 0.1395 | 4 | A1A102536.1 | α-galactosidase | GH27 | 0.38 | Y |
| 0.1517 | 11 | A1A106294.1 | β-galactosidase | GH43 | 0.41 | Y |
| 0.0467 | 2 | A1A112236.1 | β-galactosidase | GH27 | 0.85 | Y |
| 0.0169 | 1 | A1A110919.1 | wxo-β-D-glucosaminidase | GH2 | 0.07 | Y |
| 0.2033 | 9 | A1A109621.1 | β-galactosidase/β-glucuronidase | GH2 | 1.90 | Y |
| 0.045 | 2 | A1A108170.1 | GT β-(1-3)glucanosyltransferase | GH72 | 0.52 | Y |
| 0.1035 | 4 | A1A102650.1 | β-1 3-glucanosyltransferase | GH72 | 0.84 | Y |
| 0.1763 | 11 | A1A111555.1 | α-1,2-mannosidase | GH92 | 0.97 | Y |
| 0.0956 | 4 | A1A111563.1 | α-1,2-mannosidase | GH92 | 0.66 | Y |
| 0.0329 | 1 | A1A105536.1 | Hydrolase |  | 0.81 | N |
| 0.1241 | 2 | A1A103446.1 | α/β hydrolase | GH76 | 0.34 | N |
| 0.0795 | 2 | A1A102034.1 | α/β hydrolase | GH76 | 0.57 | N |
| 0.1413 | 6 | A1A105272.1 | glucamylase with starch binding domain | GH15 | 1.45 | N |
| 0.3391 | 12 | A1A103020.1 | 4-O-methyl-glucuronoyl methylesterase |  | 1.40 | Y |
| 0.0843 | 2 | A1A102529.1 | D-xylose 1-dehydrogenase |  | 1.26 | N |
| 0.0194 | 2 | A1A100862.1 | WSC domain-containing protein |  | 1.07 | Y |
| 0.0303 | 1 | A1A106682.1 | FAD-binding domain-containing protein |  | 0.23 | Y |
| 0.0183 | 1 | A1A108253.1 | CBM13 protein | CBM13 | 0.54 | Y |
| 0.0284 | 1 | A1A100921.1 | GH24 lysozyme | GH24 | 1.37 | N |
| 0.0537 | 2 | A1A101627.1 | methyltransferase domain-containing protein |  | 0.91 | N |
| 0.030 | 1 | A1A101987.1 | α-L-fucosidase | GH3 | 2.03 | N |
| 0.1297 | 7 | A1A107784.1 | GH95 alpha-L-fucosidase | GH95 | 0.30 | Y |
| 0.1681 | 5 | A1A112210.1 | GH16 protein | GH16 | 2.04 | Y |
| 0.0185 | 1 | A1A109174.1 | WSC domain-containing protein |  | 2.03 | N |
| 0.1681 | 7 | A1A105592.1 | GH43 protein | GH43 | 1.07 | N |
| 0.0288 | 1 | A1A102386.1 | GH62 protein | GH62 | 0.35 | Y |
| 0.2140 | 3 | A1A107414.1 | Carbohydrate-binding family 9 protein |  | 0.79 | Y |
| 0.0220 | 1 | A1A112382.1 | Chitinase | GH18 | 1.33 | Y |
| 0.2810 | 5 | A1A106058.1 | GH93 protein | GH93 | 2.80 | Y |

**Table S2: PCR primers used in this study**

| **Primers** | **Sequence (5' - 3')** |
| --- | --- |
| **Primers for strains construction and verification** | |
| E-*Tgparp* | AAATTACGAAACAGTTGGAGGGTGT |
| E-*HygB*-R | CCTGGCAAAACGGTATCATCAAC |
| *Tgparp*-uF | ACCTATAGCTCTTCATCTCTCCCCT |
| *Tgparp*-uR | GCCATATTGATGTAAGGTAGCTCTCGAGCAGTAGAGGTGAATGCTGTG |
| *Tgparp*-dF | GGGTATTCCATCTAAGCCATAGTACGCGGCTACTGTGTAAAAATAGCT |
| *Tgparp*-dR | AATGATCGGTACCACTGCTCGCA |
| *Tgparp*-F | ATGCCTCGAAGAAAGGCTGC |
| *Tgparp*-R | ATCTGAACACGCAAGAGATAGCG |
| E-*Tgadprase* | CTGGGAGTTGGCGCATGTGAAA |
| *Tgadprase*-uF | TCATGCCGTAAAACACCGTTGGCG |
| *Tgadprase*-uR | GCCATATTGATGTAAGGTAGCTCTCAGAAGTGAAGAGGGGTGAGAGG |
| *Tgadprase*-F | CTCAAACTCCCAAAACAACCCACATGTCAATCGCATAGCCTTCCG |
| *Tgadprase*-dR | AAGAGGAACAGGACGCACATGC |
| *HygB*-F | GAGAGCTACCTTACATCAATATGGC |
| *HygB*-R | GGTACTATGGCTTAGATGGAATACCC |
| Promoter-F | GGGTATTCCATCTAAGCCATAGTACGAGAGAAGATGGCAGTGTAGAAGGG |
| Promoter-R | ATGTGGGTTGTTTTGGGAGTTTGAG |
| Q-ADP-F | CTCATCGACGCGGGTGAAA |
| Q-ADP-R | CGCCAACGGAACGGTAAAGA |
| *Tgura*3-F | CAGTATGGTCAACTACGGTCCAGC |
| *Tgura*3-R | CGTATCTGATCAAGGAACGTTAGCG |
| E-*Tgura*3-R | CATCCAATGCAATGCATGCGAG |
| E-*Tgire* | CCACCACCAACGTTGAAGCTTCTTA |
| *Tgire*-uF | GCATTTTAGTGAGTGAGTCAGTGCG |
| *Tgire*-uR | GCTGGACCGTAGTTGACCATACTG GGCTAGAGGCTCTACCAAGAGAG |
| *Tgire*-dF | GCTGGACCGTAGTTGACCATACTG GATATTTGTCGTGTGTCGCATGG |
| *Tgire*-dR | ATGTTTCCTCAACTGCCCTTGGA |
| *Tgire*-F | ATGATTCGACGGCCCCCG |
| *Tgire*-R | CTATAATCCAGCAGGCTCATAATAC |
| **Primers for preparation of *parp*-Probe** | |
| *Tgparp*-Probe-F | CGATGAAGAGGTCAAGATTGCGG |
| *Tgparp*-Probe-R | GGAATTCAGTGCTCTCCTTGTC |
| **Primers for quantitative RT-PCR** | |
| A1A106567.1-F | TGTTCAAATCGCCATTCCTC |
| A1A106567.1-R | GCGTTCCGCTGCCTGTAAAA |
| A1A101831.1-F | GATAGCGGCAATGCTGGTC |
| A1A101831.1-R | AGGAGGCGTGGTGGAGGTT |
| A1A107632.1-F | TTGTTCCCAACTTCCACCCT |
| A1A107632.1-R | TGCCATCATCGAAATGCTTA |
| A1A110863.1-F | CAGTGGTCCGACGAGCTGTA |
| A1A110863.1-R | TGGTGCGAGTGCTTGTGATA |
| A1A104288.1-F | TACTGGCTGGCGGCTCTATT |
| A1A104288.1-R | CCTGGTCGTTGATGCTGAAA |
| A1A110611.1-F | ACCAACTGCGATGCCACCAA |
| A1A110611.1-R | GCTGCGAGCAAACTGCCACA |
| A1A102028.1-F | GCGGCACCTCATTCACAGAC |
| A1A102028.1-R | GGTGGAAGCAGTGGCGTTTG |
| A1A104298.1-F | TTTCCGACATTCGCATCCTT |
| A1A104298.1-R | CGCATTGTAGTTAGCCACGTTAG |
| A1A104556.1-F | GGCGGCAGTGCAGGATACAA |
| A1A104556.1-R | GGCGGCAGTGCAGGATACAA |
| A1A102650.1-F | ATACGACGAGCAGACCACCTT |
| A1A102650.1-R | GTAGACGATACCACCGCTGAAA |
| A1A108865.1-F | CTTCACCTCCCAGTCCAAGTAT |
| A1A108865.1-R | GCATTGACGCAAGTGTTTCC |
| A1A110026.1-F | GGAATGTTTCAGCGAAGACC |
| A1A110026.1-R | CGACATGATAGAAGTAGCACCAC |
| A1A105994.1-F | GGCTGCGAGCAATGGAAACA |
| A1A105994.1-R | GGTGCCGTAGAGGTTGTGGG |
| A1A107778.1-F | ATACAATCCCTTGAATGAGCC |
| A1A107778.1-R | AAGCCATAGTTGGAATAGTCGTG |
| A1A111035.1-F | GCGGCAACAACGACCACTAT |
| A1A111035.1-R | ACACGGATGACCTGGAAACC |
| A1A106493.1-F | CGACTGGTGTTGCCCTTGC |
| A1A106493.1-R | CCGAGGAGAATGTAGGTCTTGGT |
| A1A111258.1-F | GCTCATGTACCAGCGTGGAG |
| A1A111258.1-R | CCCTTTGGTTTGCGTCCTAT |
| A1A102057.1-F | TGAAATGGGATGCGACTGAA |
| A1A102057.1-R | GGAAAGCGATGCGAACAAAG |
| A1A111547.1-F | GTGCTCACAGGAAGGGAATC |
| A1A111547.1-R | GCAATAGTACAGCGTCAAACCAG |
| A1A102817.1-F | CAACGCCGTGATTCAAGTCC |
| A1A102817.1-R | TCAGCATCCCAGTCACCTCC |
| A1A112191.1-F | CGGCTTCGCCAAGGCTATT |
| A1A112191.1-R | TTGACCACCACCCATCTGC |
| A1A105029.1-F | GGCACTTTCCACCTTTACCC |
| A1A105029.1-R | GTTCCCTGTGAGCAGCATTT |
| A1A100991.1-F | ATGAAGTGCCTTCGAGTACTCGCTC |
| A1A100991.1-R | GAGCGAGTACTCGAAGGCACTTCAT |
| *Tgace*1-F | GACCGCCACATCAACGACAAG |
| *Tgace*1-R | AAGGAGTAGAGACAGTGCCAAGAG |
| *Tgcre*1-F | CTATGAGCTGCCGAGTCTGAGAA |
| *Tgcre*1-R | GGAATACACCGTCTGAGAGGAGG |
| *Tgxyr*1-F | CTGCCAACGAGGACGCTGAT |
| *Tgxyr*1-R | CCGCTTGCCACTTGATGTCAT |
| *Tgire*-F2 | TCTCTGCGAACTGTACCTCAAGAT |
| *Tgire*-R2 | AGAACCGTTAGAACCATCCTGTGA |
| 4742-F | GGATCATTACCGAGTTTACAACTCC |
| 4742-R | CCGTTGTTGAAAGTTTTGATTCATTT |
| *Tef*-F | TACAAGATCGGTGGTATTGGAAC |
| *Tef*-R | AGCTGCTCGTGGTGCATCTC |
| **Primers for verifying ATP sensor** | |
| E-ATP-F | CAGACAATGATGGTAGCAGCGC |
| E-ATP-R | TCGCCAGTCCCTTGTATGTGC |
| **Primers for the construction and verification of fluorescent strains** | |
| E-GFP | AAGTCGTGCTGCTTCATGTGG |
| E-*egl* | CTATATCTTGGAGCAACTGGTGTC |
| *egl*-F | TATCACAAGCACTCGCACCACT |
| *egl*-R | ATGATGATGATGATGATGTCAACGGGCAAGGCACATCG |
| *egl*-d-F | GGTGCTGGCCATATTGATGTAAGGGAACATGATGCATTGTCTAGCAAGT |
| *egl*-d-R | ACTCGGCTCCGTCTGGAATG |
| E-*cbh* | CCAGGAGTTCACACTCTCTGGC |
| *cbh*-F | GAAGTTCATCAACGGTCAGGCC |
| *cbh*-R | ATGATGATGATGATGATGCAGGCACTGAGAGTAGAATGGGTT |
| *cbh*-d-F | GGTGCTGGCCATATTGATGTAAGGTTCTGATGCTAGCAAAGACGGTCAT |
| *cbh*-d-R | ACAGAGAACCCGGAACTGCA |
| E-*bgl* | GTGGTCGTTACCCTTATGTGGC |
| *bgl*-F | CCGGCAACGGCAACTTTT |
| *bgl*-R | ATGATGATGATGATGATGTGTGGTATAACGGCCAACATCAC |
| *bgl*-d-F | GGTGCTGGCCATATTGATGTAAGGGAAACGCGCTATGGAGATGG |
| *bgl*-d-R | ACTTCATGTGCCAGGGTGGA |
| E-*xyl* | CACCTTGGCAGGTCATAGCT |
| *xyl*-F | ATGTTGTACACAACACTCCTCTCCC |
| *xyl*-R | ATGATGATGATGATGATGCAATGCACTTATGACAGCGTTGT |
| *xyl*-d-F | GGTGCTGGCCATATTGATGTAAGGTTTCGCCAATGGAAGAGTAGTAGAT |
| *xyl*-d-R | GTGCCTGAACTATACGTAGAGGTTG |
| **Primers for the construction and verification of RNAi-*Tgparp*** | |
| Sence-*Tgparp-*F1 | TTACCTATTCTACCCAAGCATCGATATGCCTCGAAGAAAGGCTGC |
| Sence-*Tgparp-*R1 | AGGCAAGAAGAAAGGCTCACCTACATCTGAACACGCAAGAGA |
| Antisence-*Tgparp-*F2 | TTGTGAAATTGTTCAAAACACACAGCTACATCTGAACACGCAAGAGA |
| Antisence-*Tgparp-*R2 | GATTTCAGTAACGTTAAGTGGATCCATGCCTCGAAGAAAGGCTGC |
| PSilent-F | GGATCCACTTAACGTTACTGAAATC |
| PSilent-R | ATCGATGCTTGGGTAGAATAGGTAA |
| intron-F | GTGAGCCTTTCTTCTTGCCT |
| intron-R | CTGTGTGTTTTGAACAATTTCACAA |
| **Primers for quantitative RT-PCR of genes related to DNA repair** | |
| *Tgalkb*-F | CTTGGGACCCGAGTTTGC |
| *Tgalkb*-R | TGGATCATTGTGTTGAGGAGGTGGG |
| *Tgrad50*-F | GCTGCGTCAAGTGTATTCC |
| *Tgrad50*-R | TCGGGTTGCTTCTTCTTT |
| *Tgrhp54*-F | CGTCGTGCGTGGTAGATT |
| *Tgrhp54*-R | GGCTGTAGCCCAGAGTTG |
| **Primers for the construction and verification of strains expressing NAD^+^ sensor** | |
| mCherry-cpYFP-F | CTCTCTGGCTACTAGAGAACCACTG |
| mCherry-cpYFP-R | GGTGCTGGCCATATTGATGTAAGGGTTGTACTCCAGCTTGTGCCCC |
| mCherry-FiNad-F | ATGGTGAGCAAGGGCGAG |
| mCherry-FiNad-R | GGTGCTGGCCATATTGATGTAAGGTTAGCCCATCATCTCCTCCCG |

# 3. Supplementary Methods

## 3.1 Phenotype and electron microscopy analysis

For phenotypic analysis, the growth of *T. guizhouense* or mutants cultured in rice straw medium plate was recorded and photographed by DSLR camera (Canon EOS 600D camera). For electron microscopy analysis, the samples from different treatments were immediately dehydrated in 5 mL of ice-cold 200 proof ethanol (Sigma-Aldrich) after fixation with 2.5% glutaraldehyde for two days, point-dried in a critical point dryer (HCP-2, Hitachi High-Technologies Corporation, Japan), and then coated with 60% Au/Pd in a sputter coater (Sputter Coater Baltec SCD500, Bal-Tel)^1^. The surface morphologies of different samples were observed using an S-4800 II field emission scanning electron microscope (Hitachi, Japan).

## 3.2 Quality control of MS data and SWATH quantification

All spectra generated from DDA were searched against the database using ProteinPilot software^2^. The group format file results were used to build a spectral library after filtration by peptides confidence > 95%. Skyline automatically matches peptide ions and corresponding fragment ions to SWATH-MS data according to ion intensity, peak shape, and peptide retention time^3^. Then, using a novel "decoy-transition" approach, mProphet automatically adapts the error model for each dataset and assigns a confidence measure to each peak group for quality control. At last, only results with an FDR cutoff of less than 0.01 were confidently accepted. SWATH-MS data were analyzed by Skyline software (version3.5), which integrated the mProphet algorithm and effectively controlled result quality. *P*-value distribution of mProphet fitting showed that target result mainly located in range 0 to 0.01, and Decoy result was uniformly distributed, which demonstrated that analysis result of SWATH-MS data was credible. In addition, the Pearson correlation was used to compare the quantification results^4^.

## 3.3 Southern blot

Fungal strains of wide type and Δ*parp* were grown in PDA medium for three days at 28 °C. Subsequently, the mycelia were harvested and lyophilized for DNA extraction. Genomic DNA was extracted using the cetyltrimethylammonium bromide method, described previously by O'Donnell et al.^5^. According to the manufacturer's protocol, the DIG High Prime DNA Labeling and Detection Starter Kit I was used for the Southern blot analysis (Roche, Basel, Switzerland). For DNA blot analysis, the genomic DNA of wild-type and the positive plasmid with *Tgparp* ORF were digested with the restriction enzyme EcoRI and BamHI. Then, about 5 μg of e digested DNA was loaded on a 0.8 % agarose gel, and the probe location was selected upstream of gene *Tgparp*, and amplified and sequenced from genomic DNA with primers. All the primers are listed in Table. S2.

## 3.4 Biolog phenotype microarrays

Growth condition was monitored by using Biolog FF Microplates, which contained 95 different carbon sources in each well and one well with water (BIOLOG, Hayward, USA), as described by Druzhinina et al.^6^ with some modifications. Spores were harvested from the 7-day-old PDA cultures and then suspended in milli-Q water, and the concentration was adjusted to 10^7^ spores mL^-1^ using a Biolog turbidity meter at O.D. 590nm. 90 μL of the spore suspension was dispensed into each well, and the plates were incubated in darkness at 25 °C. The assays were carried out at least with three replicates per genotype, and the values of O.D.750 nm were measured 12, 18, 24, 36, 48, 60, 72, 96 post-inoculations.

**3.5 Relative expression of lignocellulase genes in wt and Δ*Tgparp***

The mycelia of wt and Δ*Tgparp* were grown on the medium containing rice straw at 37 ^o^C. For qRT-PCR, fresh hyphae were collected, and total RNA extraction was performed by using the RNeasy^®^ Plant Mini Kit (Qiagen, Germany), and cDNA synthesis was completed by using the PrimeScript RT Reagent Kit (RR036A, Takara, Dalian, China) according to the manufacturer's instructions. Lignocellulase genes were selected based on the SWATH analysis results, and higher protein abundance of the selected genes relative to others made them more representative. The qRT-PCR was performed using SYBR Premix Ex Taq II (RR820A, Takara, Dalian, China) and the CFX Connect Real-Time System (Bio-Rad, Hercules, USA). Transcription levels of the target genes were normalized by the 2^-ΔCt^ method, and the translation elongation factor 1 alpha (*Tef*) was used as the housekeeping gene. All primers used were listed in Table. S2, and at least three biological replicates were carried out for each experiment.

**3.6 Preparation of protoplasts for mutants construction**

Protoplast formation was carried out according to the method of Kitamoto et al.^7^ with slight modifications. The following three solutions were configured as required, containing 200 mL solution A (1.2 M sorbitol, and 0.1 M KH_2_PO_4_, pH 5.6), 100 mL Solution B (1 M sorbitol, and 50 mM CaCl_2_, and 10 mM Tris-HCl, pH 7.5). Afterward, 100 μL fresh spore suspension was spread evenly on PDA medium covered with cellophane, cultivated in darkness for 16-20 h at 28 ^o^C. The cellophane with freshly germinated mycelium was torn off and soaked in cell wall enzyme lysate, which was prepared by adding 0.15 g lysing enzyme (L1412, Sigma-Aldrich, USA) to 20 mL Solution A. Digested mycelium were incubated at 100 rpm, 28 ^o^C for 2 hours. Enzymolysis solution was aspirated and filtered, and the hyphae were washed with solution A, and approximately 30 mL filtrate was collected in a 50 mL sterile centrifuge tube. Finally, the filtrates were centrifuged at 2000 rpm for 10 min at 4 ^o^C. At last, the supernatant was discarded and reconstituted with 0.5 mL solution B to obtain protoplasts (about 10^8^ cell mL^-1^).

**3.7 Generation of double deletion mutant**

For targeted deletion of gene *Tgire*, the 5′ and 3′ flanking regions of *ire* were amplified by PCR from *T. guizhouense* genomic DNA. *Tgura3* was used as a screening gene, and the fragment was amplified by PCR from *T. guizhouense* genomic DNA. The three fragments were fused by CloneAmp HiFi PCR Premix (Takara, Japan) according to the manufacturer’s instructions. The *Tgire* deletion mutant was generated via a gene replacement strategy based on Δ*Tgura3* deletion mutant using the polyethylene glycol (PEG)-mediated protoplast transformation procedure as described in Zhang et al^8^. For the generation of double deletion mutant Δ*Tgparp*Δ*Tgire*, the 5′, 3′ flanking regions of *Tgparp*, and the *HygB* fragments were fused and then transformed into Δ*Tgire* mutant. All the primers were listed in Table. S2.

**3.8 Observation of adding exogenous ATP-Na_2_**

ATP-Na_2_ with purity greater than 99% (Beyotime, China) was dissolved and adjusted to different concentrations. Dilute solutions were filtered through a 0.22 μm millipore filter membrane (Merck Millipore, German) to remove the impurities before being added to the rice straw medium. The wild type was cultured in rice straw medium plate at 37 °C for 48 h, and the growth condition was recorded.

**3.9 Generation of RNAi-***Tg****parp* mutants**

The pSilent-1 is a shuttle plasmid carrying resistance genes for hygromycin and ampicillin. It contains two multicloning sites flanking the intron of the Phytophthora infestans cutinase gene. To obtain the *Tgparp* gene inhibition strains in *T. guizhouense*, the sense and antisense strand fragments of *Tgparp* were amplified by PCR from genomic DNA and inserted into MCS1 and MCS2 positions in pSlient-1, respectively, by using the ClonExpress-II One Step Cloning Kit (Vazyme Biotech, Nanjing, China). The constructed vector of pSilent-*Tgparp* was transformed into *T. guizhouense* using the polyethylene glycol (PEG)-mediated protoplast transformation procedure^8^. The vector can express the stem-loop structure RNA that could be recognized and cut by Dicer to form a Silencing-complex (RISC). RISC could specifically identify and cut the RNA transcribed by the target gene. Transformants were selected by PCR and qRT-PCR, and two transformants at least were used as follow-up experiments. All the primers were listed in Table. S2.

**3.10 Generation of strains expressing the NAD^+^ sensor**

The entire coding sequence for mCherry-FiNad or mCherry-cpYFP was obtained according to Zou et al.^9^, and the corresponding fragments were synthesized and fused with *HygB* by CloneAmp HiFi PCR Premix (Takara, Dalian, China) according to the manufacturer’s instructions. The recombinant fragments were transformed into wt and Δ*Tgparp* strain using the polyethylene glycol (PEG)-mediated protoplast transformation procedure^8^. All the primers were listed in Table. S2.

## 3.11 Confocal imaging and fluorescence intensity analysis of NAD^+^ sensor

The mycelia were grown on the solid medium containing rice straw at 37 ^o^C. For confocal imaging of NAD^+^ sensor, fresh hyphae of different mutants with NAD^+^ sensor were recorded on a confocal fluorescence microscope (TCS SP8, Leica, Germany) by using a 60x water immersion objective, and the channel of mCherry was excited at 561 nm and emitted at 570 nm, while cpYFP was excited at 488 nm and emitted at 500 nm. The fluorescence intensity analysis were performed by plot profile in ImageJ software as previously described by Zhao et al.^10^.

# References

1. Wang, M. *et al.* Insights on the aerobic biodegradation of agricultural wastes under simulated rapid composting conditions. *Journal of Cleaner Production* **220**, 688-697 (2019).

2. Frederick, K. & Ciborowski, P. SWATH-MS: Data Acquisition and Analysis. in *Proteomic Profiling and Analytical Chemistry* 161-173 (Elsevier, 2016).

3. Schubert, O.T. *et al.* Building high-quality assay libraries for targeted analysis of SWATH MS data. *Nature protocols* **10**, 426-441 (2015).

4. Hack, C.J. Integrated transcriptome and proteome data: the challenges ahead. *Briefings in Functional Genomics* **3**, 212-219 (2004).

5. O'Donnell, K., Cigelnik, E., Weber, N.S. & Trappe, J.M. Phylogenetic relationships among ascomycetous truffles and the true and false morels inferred from 18S and 28S ribosomal DNA sequence analysis. *Mycologia* **89**, 48-65 (1997).

6. Druzhinina, I.S., Schmoll, M., Seiboth, B. & Kubicek, C.P. Global carbon utilization profiles of wild-type, mutant, and transformant strains of *Hypocrea jecorina*. *Applied and Environmental Microbiology* **72**, 2126-2133 (2006).

7. Kitamoto, Y., Mori, N., Yamamoto, M., Ohiwa, T. & Ichikawa, Y. A simple method for protoplast formation and improvement of protoplast regeneration from various fungi using an enzyme from *Trichoderma harzianum*. *Applied microbiology and biotechnology* **28**, 445-450 (1988).

8. Zhang, J. *et al.* Guttation capsules containing hydrogen peroxide: an evolutionarily conserved NADPH oxidase gains a role in wars between related fungi. *Environmental microbiology* **21**, 2644-2658 (2019).

9. Zou, Y. *et al.* Illuminating NAD^+^ metabolism in live cells and in vivo using a genetically encoded fluorescent sensor. *Developmental cell* **53**, 240-252. e7 (2020).

10. Zhao, X. *et al.* Elaiophylin, a novel autophagy inhibitor, exerts antitumor activity as a single agent in ovarian cancer cells. *Autophagy* **11**, 1849-1863 (2015).

1. * To whom correspondence should be addressed: [liudongyang@njau.edu.cn](mailto:liudongyang@njau.edu.cn); Tel: +86 25 84396853; Fax: +86 25 84396853, 1. Key lab of organic-based fertilizers of China and Jiangsu provincial key lab for solid organic waste utilization; 2. Nanjing Agricultural University, Nanjing 210095, Jiangsu, China. [↑](#footnote-ref-1)
